# Treating a type 2 diabetic patient with impaired pancreatic islet function by personalized endoderm stem cell-derived islet tissue

**DOI:** 10.1038/s41421-024-00662-3

**Published:** 2024-04-30

**Authors:** Jiaying Wu, Tuo Li, Meng Guo, Junsong Ji, Xiaoxi Meng, Tianlong Fu, Tengfei Nie, Tongkun Wei, Ying Zhou, Weihua Dong, Ming Zhang, Yongquan Shi, Xin Cheng, Hao Yin, Clinical Group, Xiaoyu Mou, Yifan Feng, Xiaoliang Xu, Junfeng Dong, Duowen He, Yuanyu Zhao, Xue Zhou, Xueqi Wang, Feng Shen, Yue Wang, Guoshan Ding, Zhiren Fu

**Affiliations:** 1 Shanghai Institute of Biochemistry and Cell Biology, Center for Excellence in Molecular Cell Science, Chinese Academy of Sciences Shanghai, China; 2 Department of Endocrinology, Shanghai Changzheng Hospital (Second Affiliated Hospital of Naval Medical University), Shanghai, China; 3 National Key Laboratory of Medical Immunology & Institute of Immunology, Naval Medical University, Shanghai, China; 4 Organ Transplant Center, Shanghai Changzheng Hospital, Shanghai, China; 5 Department of Interventional Radiology, Shanghai Changzheng Hospital, Shanghai, China; 6 Department of Urology, Renji Hospital, School of Medicine, Shanghai Jiao Tong University Shanghai, China; 7 Islet Transplantation Training Base of Shanghai Endocrinology Clinical Quality Control Center, Shanghai, China; 8 Department of Health Care, Department of Translational Medicine, Shanghai Changzheng Hospital, Shanghai, China; 9 Department of Hepatic Surgery IV, Third Affiliated Hospital of Naval Medical University (Eastern Hepatobiliary Surgery Hospital), Shanghai, China; 10 Shanghai Key Laboratory of Cell Engineering, Research Center of Translational Medicine, Naval Medical University, Shanghai, China

**Keywords:** Stem cells, Stem-cell differentiation, Regeneration

Dear Editor,

Type 2 diabetes (T2D) typically starts with insulin resistance in peripheral tissues and proceeds with gradual loss of islet function due to the reduction in β-cell mass or dedifferentiation of β cells^1,2^. More than 30% of T2D patients eventually rely on exogenous insulin treatment. Cadaveric islet transplantation is an effective treatment for insulin-dependent diabetes^3,4^. Notably, improved metabolic control after islet transplantation is associated with better kidney allograft function and long-term survival^5,6^. However, the application of islet transplantation is severely hampered due to the critical shortage of donor organs.

The pancreatic progenitor (PP) cells or islet tissues, generated from human pluripotent stem cells (hPSCs), have been shown to survive, function and reverse hyperglycemia in diabetic animal models^7–9^. In addition, a recent clinical trial has shown that, when subcutaneously implanted into T1D patients, the hPSC-derived pancreatic endodermal cells encapsulated with non-immunoprotective devices were able to further mature into meal-responsive β-like cells and secrete insulin, albeit at the levels insufficient to achieve the independence of exogenous insulin^10,11^. Nevertheless, clinical applications of hPSC-derived cells are undermined by the complicated differentiation processes and the risk of having residual undifferentiated cells that may form teratomas in vivo. Recent studies have focused on identifying intermediate stem cell types, including the non-tumorigenic human endoderm stem cells (EnSCs)^12^, which appear to be more suitable as precursors for large-scale generation of islet cells.

**Fig. 1:**
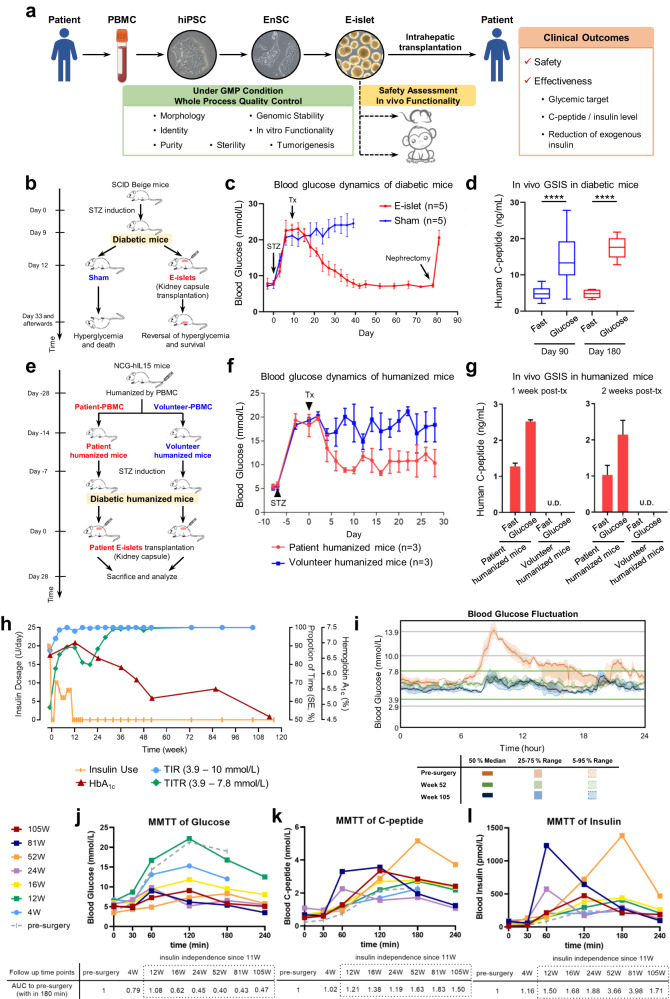
Preclinical studies and clinical outcomes of autologous E-islet transplantation in a T2D patient. **a** Brief scheme of major procedures involved in the generation and quality control of E-islets and the safety/effectiveness evaluations of E-islet transplantation. **b**–**d** E-islets reverse hyperglycemia in STZ-induced diabetic immunocompromised mice. Schematic illustration of kidney capsule transplantation of E-islets (**b**). Fasting blood glucose dynamics (blue line: sham group; red line: E-islet-transplanted group, **c**). Secretion of human C-peptide after fasting and 30 min following an i.p. glucose bolus on days 90 and 180 post transplantation (**d**). **e**–**g** Immunogenicity of E-islets in humanized mice. Schematic illustration of the syngeneic and allogeneic kidney capsule transplantation of patient-specific E-islets into the NCG-hIL15 diabetic mice humanized with the patient’s and a volunteer’s PBMCs (**e**). Fasting blood glucose dynamics (blue line represents the control group with the patient E-islets transplanted into three diabetic mice humanized with the volunteer’s PBMCs; red line represents the group with the patient E-islets transplanted into three diabetic mice humanized with the patient’s PBMCs, **f**). Secretion of human C-peptide after fasting and 30 min following an i.p. glucose bolus on days 7 and 14 post E-islet transplantation (U.D. undetectable, **g**). **h** Clinical measurements of TITR, TIR and HbA1c, and the insulin dosage during 116 weeks. **i** Continuous interstitial glucose fluctuations derived from the CGM measurements at weeks 52 and 105 compared with pre-surgery levels. **j**–**l** Serum levels of fasting and meal-stimulated circulating glucose (**j**), C-peptide (**k**) and insulin (**l**) from MMTT assays.

Here, we report the intrahepatic implantation of islet tissue (E-islets) differentiated in vitro from autologous EnSCs in a T2D patient who had impaired insulin secretion. This is a pilot study of an investigator-initiated trial designed to investigate the safety and efficacy of E-islets for the treatment of insulin-dependent diabetic patients (Fig. 1a). The patient was a 59-year-old man with a 25-year history of T2D who developed end-stage diabetic nephropathy and underwent kidney transplantation in June of 2017 and displayed poor glycemic control since November of 2019, characterized by blood glucose level ranging from 3.66–14.60 mmol/L, mean amplitude of glycemic excursion (MAGE) of 5.54 mmol/L, the time-in-the-tight-target-range (TITR, 3.9–7.8 mM) of 56.7%, with daily hyperglycemic events (> 10.0 mmol/L) of 0.7/d and hypoglycemic events (< 3.9 mmol/L) of 0.3/d (Supplementary Table S1). Due to the major concerns of hypoglycemia and the detrimental effect of poor glycemic control on the long-term survival of the donor kidney, the patient agreed to pursue transplantation with autologous E-islets.

E-islets were generated from the autologous EnSCs that are established under the culture condition modified from our previous reports^12^ (see details in Supplementary methods), through two intermediate stages under GMP conditions. The morphology, purity, viability and microorganism contamination of EnSC-derived PPs, endocrine progenitor cells and E-islets were proven to meet the release criteria (Supplementary Figs. S1–S3 and Table S6). E-islets displayed similar morphology (Supplementary Fig. S3a), endocrine cell composition (Supplementary Fig. S3b, c, e, f), gene expression patterns (Supplementary Fig. S3d–f) and in vitro functionality (Supplementary Fig. S3g) to human cadaveric islets, and showed functional efficacy in Streptozotocin (STZ)-induced diabetic mouse (Fig. 1b–d) and monkey (Supplementary Fig. S4) models. The nontarget hepatic or intestinal lineages, when examined by either scRNA-seq (Supplementary Fig. S3f) or FACS (Supplementary Fig. S3h), were not detected. Neither tumor formation nor cystic/ductal structures that indicate cell proliferation were detected in the immunocompromised animals transplanted with either EnSCs or E-islets during the experiments (Supplementary Table S3). The patient-specific E-islets survived and functioned under the kidney capsules of the diabetic immunocompromised mice humanized with patient’s own PBMCs, but rejected by the ones humanized with PBMCs from an unrelated volunteer (Fig. 1e–g; Supplementary Fig. S5), which suggests that patient’s immune system likely tolerates the autologous E-islets.

The patient underwent a percutaneous transhepatic portal vein transplantation with 1.2 million IEQs of E-islets delivered, conforming to the regulatory guidance from the clinical islet transplantation registration. At designated visits, examinations of endocrine function and diabetes-specific parameters by mixed-meal tolerance test (MMTT) were performed at baseline, 4, 8, 12, 16, 20, 24, 36 and 48 weeks and thereafter at specified time points (Supplementary Fig. S6a). The glycemic control of the patient was measured with a 24-h real-time continuous glucose monitoring system (CGM).

During the 116-week follow-up period, no tumor formation was detected either by MRI on the upper abdomen or by the measurements of serum tumor-related antigen markers. The treatment-emergent adverse events included: (1) temporary abdominal distension and loss of appetite within 4–8 weeks, relieved with methionyltrichloride; (2) restorable weight loss < 5% (from 80 kg to 76 kg).

The three major clinical outcomes, the glycemic targets, the reduction of exogenous insulin and the levels of fasting and meal-stimulated circulating C-peptide/insulin were monitored throughout the first 116 weeks (Supplementary Tables S4, S5). Marked changes in the patient’s glycemic control were observed as early as week 2 post transplantation, as the MAGE declined from 5.50 mmol/L to 3.60 mmol/L, and the TITR increased rapidly from 56.7% to 77.8% (Fig. 1h; Supplementary Table S1). Over the same period, the time-above-range (TAR) decreased by 55% from baseline, while the events of severe hyperglycemia (> 13.9 mM) and hypoglycemia (< 3.9 mM) completely disappeared (Supplementary Fig. S7a, b and Table S1). During the period between weeks 4 and 12, a significant reduction in ambulatory mean glucose fluctuations (from 5.50 to 2.6 mmol/L) (Supplementary Table S1) and a steady rise in TITR (from 81% to 90%) were observed (Fig. 1h; Supplementary Fig. S7c–e and Table S1). After week 32, the patient’s TITR had readily reached 99% and was maintained thereafter (Fig. 1h, i; Supplementary Table S1), while MAGE, the gold standard of blood glucose variability, was reduced from 5.50 mM to 1.60 mM (Supplementary Figs. S6d, S7h–l and Table S1). Importantly, no episodes of hypoglycemia or severe hyperglycemia were observed during the whole follow-up period of 116 weeks post surgery (Supplementary Fig. S7 and Table S1). Additionally, MMTT revealed a trend of stabilization in glycemic variability after surgery, as manifested by the stable fasting glucose concentrations and the significant reductions in the post-meal glucose concentrations (maximum of 21.3 mM at baseline vs maximum of 9.1 mM at week 105) (Fig. 1j; Supplementary Table S5). Consistently, the area under the curve (AUC) derived from the 5-point intravenous glucose values decreased to 40% of baseline (Supplementary Fig. S6b), confirmed by the AUCs of the values acquired from CGM (Supplementary Fig. S6c). The hemoglobin A1c levels decreased from 6.6% (baseline) to 5.5% (week 85) and 4.6% (week 113) (Fig. 1h; Supplementary Table S1).

Notably, the insulin requirements were reduced gradually until complete withdrawal at the end of week 11 (Fig. 1h), and the oral antidiabetic medications were tapered since week 44 and discontinued at weeks 48 (acarbose) and 56 (metformin) (Supplementary Fig. S6a).

The average post-surgery fasting C-peptide level (0.68 nmol/L) increased by 3-fold when compared to pre-surgery level (Fig. 1k; Supplementary Table. S5). Notably, the secretions of C-peptide (Fig. 1k) and insulin (Fig. 1l) measured by MMTT revealed significant elevations compared to those of the pre-surgery tests, as confirmed by the AUCs (Supplementary Fig. S6b).

Collectively, we report the first-in-human tissue replacement therapy using autologous E-islets for a T2D patient with impaired islet function. The first 27-month data revealed significant improvements in glycemic control, and provided the first evidence that stem cell-derived islet tissues can rescue islet function in late-stage T2D patients. The grafts were well tolerated with no tumor formation or severe graft-related adverse events.

The precedent clinical trials using cadaveric islets or encapsulated hPSC-derived PPs^10,11^, along with our study, have provided encouraging evidence that islet tissue replacement is an effective cure for diabetic patients. Notably, the derivation of islet tissues from either hPSCs or EnSCs provides unprecedented new sources for tissue-replacement therapy. Despite the common proof-of-concept purpose, there are some distinctions among the published trials^10,11^ and ours. First, EnSC-based islet regeneration system is unique, in that EnSCs are nontumorigenic in vivo^12^ and amenable for efficient mass production of islets as they are endoderm-specific and developmentally closer to pancreatic lineages. Second, our pilot study chose a T2D rather than T1D patient, which not only precluded the interference from autoimmune conditions for the assessment of engraftment and functionality of E-islets but also extended the scope of indications for islet transplantation. As for the limitations of this study, we cannot completely rule out the possibility that the residual endogenous islets benefitted from the surgery and acquired functional improvements. Therefore, an increase in sample size and additional trials of T1D patients with complete loss of islet β cells will help draw definitive conclusions on the causative role of E-islets in the achievement of glycemic targets.

Future studies are warranted to address the pharmacodynamics of stem cell-derived islets as a drug, to extend the application of stem cell-derived islet transplantation to other subtypes of diabetes, and to generate “universal islets” as off-the-shelf products to cure diabetes without the need for immunosuppression.

### Supplementary information

Supplementary Information
