# Supplementary material for: Treating a type 2 diabetic patient with impaired pancreatic islet function by personalized endoderm stem cell-derived islet tissue
Source: Cell Discov. 2024 Apr 30;10:45. doi: 10.1038/s41421-024-00662-3 (PMC11058776; doi:10.1038/s41421-024-00662-3)
Supplement: Supplementary file 1 — Supplementary Information [file 41421_2024_662_MOESM1_ESM.pdf]

Treating a type 2 diabetic patient with impaired pancreatic islet function by personalized endoderm stem cell-derived islet tissue

Jiaying Wu, Tuo Li, Meng Guo, Junsong Ji, Xiaoxi Meng, Tianlong Fu, Tengfei Nie, Tongkun Wei, Ying Zhou, Weihua Dong, Ming Zhang, Yongquan Shi, Xin Cheng, Hao Yin and Clinical Group

Table of contents

Supplementary Methods **3**

Study oversight 3

Experimental resources 6

Method details 7

Quality control methods 9

Animal studies 12

Clinical studies 15

Supplementary Fig. S1. Quality control of EnSCs **17**

Supplementary Fig. S2. Quality control of intermediate pancreatic differentiation stages of EnSCs **19**

Supplementary Fig. S3. Quality control of E-islets **22**

Supplementary Fig. S4. E-islets ameliorate diabetes in STZ-induced diabetic monkeys 23

Supplementary Fig. S5. Characterization of humanized mice **26**

Supplementary Fig. S6. Clinical assessments and outcomes of glycemic control **27**

Supplementary Fig. S7. Continuous glucose monitoring at various follow-up time points **30**

Supplementary Table Information **31**

Supplementary Table S1. Exploratory objectives **31**

Supplementary Table S2. Summary of SNV discovered from whole genome sequencing of

EnSCs compared to parental PBMCs......32

Supplementary Table S3. Teratoma formation assay......37

Supplementary Table S4. Key laboratory values before and after transplantation......38

Supplementary Table S5. Primary follow-up objectives......39

Supplementary Table S6. Quality control release criteria of EnSCs and E-islets......40

Supplementary Table S7. Primer list......42

Supplementary Table S8. Antibody list......42

Supplementary Methods

Study oversight

This is pilot study of an investigator-initiated trial (ClinicalTrials.gov (NCT05294822)) designed to investigate the safety and efficacy of E-islets generated from autologous EnSCs for the treatment of insulin-dependent diabetic patients with impaired islet function (Fig. 1a). Informed consent included a review of available therapeutic options and a list of potential risks associated with the first-in-human use of this method. The study was approved by the ethics committee of Shanghai Changzheng Hospital [CZEC(2019)-03] and conducted under regulatory guidance from the National Medical Products Administration (NMPA) and National Health Committee (NHC) (Administrative Measures for Clinical Research of Stem Cells (Trial), Quality Control of Stem Cell Preparations and Guiding Principles of Preclinical Research (Trial) and Measures for the Administration of Clinical Research Initiated by Investigators in Medical and Health Institutions). Written informed consent was obtained from participant before enrollment. All animal procedures were conducted with approval from the CAS Center for Excellence in Molecular Cell Science, Chinese Academy of Sciences. All the authors vouch for the accuracy and completeness of the data reported and the adherence of the study to the protocol, which are available with the full text of this article.

Creation of autologous EnSC

PBMCs were harvested, tested for microorganisms and used to generate iPSC lines under GMP conditions. Two iPSC clones were selected for further characterization and establishment of EnSC lines (modified from reference # 12) under GMP conditions (Supplementary Fig. S1). Whole-genome sequencing (WGS) was performed on EnSC lines to confirm the absence of newly emerged cancer or diabetes-associated mutations that were not detected in the original source PBMCs (Supplementary Fig. S1 and Supplementary Table S2). EnSCs and their derivatives were tested in immunocompromised mice (SCID Beige) for tumor-forming potential for 6 months (Supplementary Table S3).

Characterization of islets derived from EnSCs

Three batches of E-islets were generated through two intermediate (pancreatic progenitor / PP and endocrine progenitor / EP) stages under GMP conditions at scales that met the dose requirement for each patient (1.2×10^6 islet equivalents [IEQs] / patient). The EnSC-derived PPs, EPs and E-islets were evaluated for microorganism contamination, morphology, purity and viability. E-islets were analyzed for endocrine cell composition (Insulin+NKX6-1+ β cells; Glucagon+ α cells; Somatostatin+ δ cells; Chromogranin A+ endocrine cells) by flow cytometry and single-cell transcriptomic analyses (scRNA-seq). The in vitro functionality of E-islets was assayed by glucose-stimulated insulin secretion (GSIS) as human cadaveric islets, and the in vivo functionality was evaluated by kidney capsule or hepatic portal transplantation into the streptozotocin-induced diabetic mouse or monkey models. The nontarget hepatocytes (HNF4A+Albumin+), cholangiocytes (SOX9+CK7+), intestinal epithelial cells (CDX2+) and the pancreatic ductal cells (SOX9+PTF1A+PDX1+) were estimated from the scRNA-seq data.

Safety monitoring

Safety endpoints included treatment-emergent adverse events (TEAEs), early discontinuation of treatment due to adverse events and adjudicated adverse events. The tumor formation was monitored every three months by enhanced magnetic resonance imaging performed on upper abdomen and by measurements of serum cancer-related antigens.

Assessments of clinical outcomes

At designated visits, the patient was weighed and reported his Clark hypoglycemia awareness score, and was subjected to routine and disease-specific assessments. Examinations of endocrine function and diabetes–specific parameters by mixed-meal tolerance tests (MMTT) were performed at baseline and at 4, 8, 12, 16, 20, and 24 weeks and thereafter every 12 weeks (Supplementary Fig. S6a). The glycemic control

of the patient was measured with a 24-hour real-time blood glucose monitoring system (Medtronic GuardianTM Connect Subcutaneous Continuous Glucose Monitoring System/CGMS). All information from the CGMS device was centrally assessed. The baseline and follow-up CGM glucose values were measured throughout the first 52 weeks, and the mean duration of CGM device wearing was at least 3 days.

As the three major clinical outcomes, the glycemic targets, the levels of fasting and meal-stimulated circulating C-peptide/insulin and the reduction of exogenous insulin were monitored throughout the first 116 weeks. A list of follow-up assessments is provided in Supplementary Tables S4 and S5.

Case report

The patient was a 59-year-old man with a 25-year history of T2D who developed end-stage diabetic nephropathy and underwent kidney transplantation in June of 2017. His estimated glomerular filtration rate (eGFR) and serum creatinine (SCr) level were maintained at 90 to 105 ml/(min·1.73 cm^2) and 45-72 μmol/L, respectively, indicating good survival and functioning of the donor organ. He had been receiving anti-rejection drugs (tacrolimus 1 mg bid and mycophenolate mofetil 0.5 mg bid) and subcutaneous insulin injection at a dose of 20 U once daily at bedtime and oral antidiabetic medications (acarbose 50 mg tid and metformin 0.75 g bid) (Supplementary Fig. S6a). However, he reported poor glycemic control since November 2019, characterized by an average blood glucose level of 7.8 ± 2 mmol/L, the time-in-the-range (TIR, 3.9-10.0 mM) of 87.7% and the time-in-the-tight-target-range (TITR, 3.9-7.8 mM) of 56.7%, with daily hyperglycemic events (> 10.0 mmol/L) of 0.7/d and hypoglycemic events (< 3.9 mmol/L) of 0.3/d. Due to the major concerns of hypoglycemia induced by insulin administration, the adverse effect of antirejection drugs on glycemic control, and the detrimental effect of poor glycemic control on the long-term survival of the donor kidney, the patient and the study team agreed to pursue transplantation with autologous E-islets. The patient underwent a percutaneous transhepatic portal vein transplantation with 1.2 million IEQs delivered, conforming to the regulatory guidance from the clinical islet transplantation registration (CITR). Portal venography and portal vein

pressure were monitored throughout the whole procedure to ensure that there was no portal embolization or portal hypertension. No glucocorticoids were used at any time. After the surgery, the patient was monitored overnight and was allowed out of bed the following day. Patient compliance with scheduled appointments was 100% (some visits were either cancelled or relocated to local hospitals due to the COVID-19 pandemic).

Experimental resources

Cell resource of human endoderm stem cell

Human endoderm stem cell lines were generated from patient-specific hiPSCs and maintained in serum-free and sromal-free conditions. The hiPSCs were generated from human peripheral blood mononuclear cells (PBMCs) of T2D patient with Sendai virus reprogramming kit (Invitrogen) containing four reprogramming factors (*OCT4, SOX2, KLF4, L-MYC*).

Human primary islets

Human primary islets were provided and isolated by Shanghai Changzheng Hospital.

Rodent strains

SCID Beige mice were obtained from Shanghai Lingchang Biotech company. All experiments were performed in accordance with protocols approved by the Institutional Animal Care and Use Committee at Shanghai Institute of Biochemistry and Cell Biology.

NCG-hIL15 mice were obtained from GemPharmatech Co., Ltd.

All animals were males and were housed in individually ventilated cages (IVC) in specific pathogen-free (SPF) animal facility with temperature and light controlled (12-h light/dark cycle).

Cynomolgus monkey model

Cynomolgus monkeys were obtained and housed in WUXI Biologics company. All animals were housed in a separate stainless-steel cage with temperature (18-26 ºC),

relative humidity (40-70 %) and light (12-h light/dark cycle) controlled. All animals provided with a continuous water supply and were fed a regular primate diet supplemented with fresh fruits twice daily (9-11 a.m. and 3-4 p.m.). All animal care and handling were performed in accordance with the guidelines established by IACUC at WUXI Biologics company.

Method details

Generation of induced pluripotent stem cells (iPSCs) from patient's peripheral blood mononuclear cells (PBMCs)

PBMCs were harvested and tested for microorganisms, including bacteria, fungi, mycoplasma, HIV, HAV, HBV, HCV, HTLV, EBV, HCMV and TP, and then used to generate iPSC lines with the Sendai Viral reprogramming system under GMP conditions. PBMCs of T2D patient (WB) were isolated from whole blood using Ficoll gradient. The whole blood was sampled with BD Vacutainer® EDTA Tubes. The blood was diluted with DPBS and poured onto the Ficoll solution, and were centrifuged for 30 minutes at 400 g at room temperature. The layer of PBMCs was collected and washed with DPBS. PBMCs were frozen or proceeded to iPSC generation. Sendai virus reprogramming kit (Invitrogen, GMP grade) containing four reprogramming factors (*OCT4, SOX2, KLF4, L-MYC*) was used for iPSC generation. Two million PBMCs were first cultured in the presence of human SCF, FLT-3, IL-3 and IL-6 in SP34 for 7 days as instructed. On the day of transduction, PBMCs were washed and counted, and the appropriate volumes of virus vectors were calculated according to the cell count and the titers of virus. The PBMCs and virus vectors were mixed for the transduction. Two days post transduction, cells were plated onto culture dishes and gradually transitioning cells to mTeSR1 medium in the next 7 days. iPSC colonies were picked and transferred onto individual culture plates around 2-3 weeks, and were maintained in a 37°C incubator with a 5% CO_2, 5% O_2, 90% N_2 environment. Ten iPSC clones at Passage 10 were tests for in vitro differentiation potential, and two of them (designated WB20 and WB34) were selected for further characterization and the establishment of EnSC lines under GMP conditions.

Generation of EnSCs from hiPSCs

EnSC lines were established from patient-specific hiPSC lines WB20 and WB34, and maintained in a 37°C incubator with a 5% CO2, 5% O2, 90% N2 environment. Endodermal cells were differentiated from hiPSCs by dual activation of Nodal and WNT signaling pathways with Activin A (100 ng/mL) and CHIR99021 (2 μM) for 24 hours, and then cultured in the presence of bFGF (5 ng/mL), Activin A (100 ng/mL), VEGF (10 ng/mL) ascorbic acid (0.5 mM, Wako) and glutaMAX (2 mM, Invitrogen) for 4 days, followed by the culturing in the presence of bFGF (10 ng/ml), TGF-a (20 ng/mL), VEGF (10 ng/mL), BMP4 (50 ng/mL), HGF (25 ng/mL), dexamethasone (40 ng/mL, Sigma) for 4 days. Thereafter EnSCs were established by replating endodermal cells at $1x10^6$ cells per milliliter and maintaining in MCDB131 supplemented with Wnt3A (1 μM), Rspondin1 (50 ng/mL), EGF (20 ng/mL), A83-01 (0.5 mM), ascorbic acid (0.5 mM, Wako) and glutamine (2 mM, Corning). EnSCs were harvested every 3-4 days and dissociated into single cells for subsequent expansion, subcloning or differentiation. Typically, EnSCs at passage 20 were selected for quality control tests including those of morphology, viability, purity, sterility, karyotype, as well as whole genome sequencing. WB20 EnSC line was finally selected as a clinical grade clone as it was devoid of known cancer-related mutations and with the lowest overall mutational burden compared to patient PBMC. EnSCs and their derivatives were tested in immunocompromised mice (SCID Beige) for tumor-forming potential for 6 months of observation.

Scalable differentiation of EnSCs into E-islets

EnSCs were thawed and expanded with T225 flasks at a starting concentration of $2x10^6$/flask. Sterility was tested before the initiation of differentiation. For the induction of pancreatic endoderm (1st stage), EnSCs were treated in MCDB with a cocktail containing LDN-193189 (200 nM), Noggin (20 ng/mL), ActivinA (0.5 ng/mL) FGF10 (20 ng/mL) Rspondin1 (20 ng/mL), EGF (20 ng/mL) and TPPB (500 nM) for 2 days; during day 2-4 of induction, cells were further differentiated in MCDB supplemented

with LDN-193189 (200 nM), FGF10 (20 ng/mL), EGF (20 ng/mL), SANT1 (0.5 μM), ascorbic acid (0.5 mM) and retinoic acid (2 μM); during day 4-6 of differentiation, cells were cultured in the presence of FGF10 (50 ng/mL), EGF (20 ng/mL), SANT1 (0.3 μM), retinoic acid (0.2 μM), Nicotinamide (10 mM) and ascorbic acid (0.5 mM). At the end of this stage, pancreatic progenitor (PP) cells were single-cell dispersed and suspended in AggreWell (STEMCELL) to form homogeneous cell clusters for 3 days and then transferred to orbital shakers (90~110 rpm) for further islet tissue reconstruction and maturation. For endocrine progenitor (EP) induction (3rd stage), triple inhibition of BMP, TGF-β and Notch signaling pathways was manipulated for 10 days in the presence of Noggin (20 ng/mL), A83-01 (0.5 mM), gamma-secretase inhibitor XX (2 μM, MERCK), retinoic acid (0.1 μM) and SANT1 (0.1 μM). For the maturation of endocrine cells (4th stage, 8-10 days), T3 (1 μM), Nicotinamide (10 mM) and BMP4 (2 ng/mL) were added to the 3rd stage recipe. MCDB was routinely supplemented with glucose (22.5mM, Sigma), sodium bicarbonate (Sigma) and ITS-X (Invitrogen), GlutaMAX (Invitrogen) and ascorbic acid (0.5 mM, Wako). All cytokines were purchased from R&D Systems, with GMP grade if applicable.

Release criteria

Release criteria is provided in Supplementary Table S6.

Quality control methods

RNA extraction and quantitative real-time PCR

The reverse transcription and qRT-PCR reactions were performed as reported previously (Cheng et al., 2012). The RNAs was prepared with an RNA kit (TIANGEN) according to the manufacturer's directions and reverse-transcribed into cDNAs using random hexamers and oligo (dT) primers with GoScript Reverse Transcriptase (Promega). The qRT-PCR reactions were performed using an ABI Q6 (Life) system and SYBR Green Master Mix (Roche). The expression levels were normalized to the housekeeping gene *TBP*. The primer information is provided in Supplementary Table

S7.

Flow cytometry

Cell samples were collected as single cells. The staining of surface markers was performed in PBS with 0.2% BSA. The cells were incubated with antibodies for 30 minutes on ice. For intracellular proteins, cells were fixed with 1.6% PFA at 37 ºC for 30 minutes and washed with the Permeabilization Wash buffer (BioLegend). The antibodies were incubated for 30 minutes at RT. Finally, cells were analyzed using a flow cytometer Celesta or Fortessa (BD). For cell viability test, calcein blue dye (Invitrogen) was used to mark live cell. Incubate cells and analyze using a flow cytometer Celesta or Fortessa (BD). See antibody information in Supplementary Table S8.

Immunofluorescence

E-islets were fixed with 4% PFA for 15 minutes at 4 ºC and permeabilized with 0.5% Triton-100 before blocking. E-islets were washed three times with PBST (0.05% Tween 20 in PBS) for 10 minutes at room temperature (RT) both before and after each staining step, and blocked with 2% BSA at 4 ºC for 2 hours. E-islets were stained with diluted primary antibodies at 4 ºC overnight, and the samples were then incubated in the diluted secondary antibodies for 2 hours at 4 ºC. All antibodies were diluted in 2% BSA in PBS. Prolong Gold Antifade reagent with DAPI (Invitrogen) was used to counterstain the nuclei. E-islets were analyzed using confocal fluorescence microscopes (Olympus FV3000). The images of E-islets were captured and 3D-projected using the Olympus software. The antibody information is listed in Supplementary Table S8.

In vitro static glucose stimulated insulin (C-peptide) secretion assay

Before glucose stimulation, E-islets or primary islets were rinsed and starved in Krebs-Ringer buffer supplemented with 2 mM glucose for 2 hours at 37 °C. For glucose stimulation, E-islets were treated alternately by Krebs-Ringer buffer with low (2 mM) or high (20 mM) glucose. Supernatants were collected after 30 minutes of each

stimulation. C-peptide was measured by a human C-peptide ELISA kit (Mercodia, 10-1141-01) according to the manufacturer's instruction.

Mycoplasma, sterility and endotoxin tests

Culture supernatant samples were sent to a certified laboratory, Shanghai Simple Gene Medical Laboratory, for testing.

Karyotype analysis

EnSC samples were sent to a certified laboratory, Shanghai Simple Gene Medical Laboratory, for standard G-banded Chromosome analysis.

Whole genome sequencing

DNA preparation

DNA degradation and contamination was monitored on 0.8% agarose gels. DNA purity was checked using the NanoPhotometer® spectrophotometer (IMPLEN, CA, USA). DNA concentration was measured using Qubit® DNA Assay Kit in Qubit® 3.0 Flurometer (Life Technologies, CA, USA).

DNA Library preparation and sequencing

The NEB Next® Ultra DNA Library Prep Kit for Illumina® (NEB， USA) was used to construct the libraries for sequencing as per the manufacturer's instructions. DNA was fragmented into ~200 base pair pieces. The end of the DNA fragment was subjected to an end repair process that included the addition of a single "A" base, followed by ligation of the adapters. Products were purified and enriched by polymerase chain reaction (PCR) to amplify the library DNA. The final libraries were quantified using KAPA Library Quantification kit (KAPA Biosystems, South Africa) and an Agilent 2100 Bioanalyzer. Paired-end sequencing (2 × 150 base pair) was performed on an Illumina NovaSeq 6000 sequencer (Illumina, USA).

Single cell RNA sequencing and data processing

Cell capture and cDNA synthesis

E-islets were dissociated into single cells with 0.25% trypsin and resuspended at 1×10^6 cells per milliliter in 1 × PBS. Using single cell 3 'Library and Gel Bead Kit V3.1(10x Genomics, 1000121) and Chromium Single Cell G Chip Kit (10x Genomics, 1000120), the cell suspension (300-600 living cells per microliter determined by Count Star) was loaded onto the Chromium single cell controller (10x Genomics) to generate single-cell gel beads in the emulsion according to the manufacturer's protocol. In short, single cells were suspended in PBS containing 0.04% BSA. About 10,000 cells were added to each channel, and the target cell will be recovered was estimated to be about 15,000 cells. Captured cells were lysed and the released RNA were barcoded through reverse transcription in individual GEMs. Reverse transcription was performed on a S1000TM Touch Thermal Cycler (Bio Rad) at 53°C for 45 min, followed by 85°C for 5 min, and hold at 4°C. The cDNA was generated and then amplified, and quality assessed using an Agilent 4200 (performed by CapitalBio Technology, Beijing).

Single cell RNA-Seq library preparation and sequencing

According to the manufacture's introduction, Single-cell RNA-seq libraries were constructed using Single Cell 3' Library and Gel Bead Kit V3.1. The libraries were finally sequenced using an Illumina Novaseq6000 sequencer with a sequencing depth of at least 30,000 reads per cell with pair-end 150 bp (PE150) reading strategy (performed by CapitalBio Technology, Beijing). 15244 cells were sequenced and 2721 cells with low UMI (UMI counts <5000) were excluded from sequenced cells during tSNE clustering analysis.

Animal studies

Teratoma formation test

SCID Beige (4~6 weeks) male mice were transplanted with 1×10^5 hPSCs or 1×10^7 EnSCs intramuscularly or cervical subcutaneously. The formation of teratoma was monitored during the period of 6 months.

Transplantation of E-islets into Streptozodocin (STZ)-induced diabetic model mice

STZ was dissolved immediately before injection in 50 mM sodium citrate buffer (pH 4.5) to a final concentration of 20 mg/mL, and kept in dark and low temperature before injection. The administrations of STZ were completed within 5 minutes after the dissolution. SCID Beige male mice (4~6 weeks) were treated with 170 mg/kg STZ (Sigma-Aldrich, S0130) by intraperitoneal injection after 4 hours of starvation. The fasting blood glucose were measured at days 5 and 8, in a tail bleed using a hand-held blood glucose meter (Roche) to ensure hyperglycemia. E-islets (1000~2000 IEQ) were transplanted under left kidney capsules of diabetic mice. Glucose-stimulated human C-peptide secretion was measured by collecting mouse serum from the eye socket after 16 hours of fasting and at 25 minutes after glucose intraperitoneal injection (3 g/kg, 30% solution).

Transplantation of E-islets into STZ-induced diabetic monkey

Induction of diabetes in monkey

To induce hyperglycemia, male Cynomolgus monkey (3~6 years) were fasted overnight and treated twice (with an interval of 2 weeks), with a dose of 50 mg/kg STZ intravenous injection. STZ was freshly dissolved before injection in sodium citrate buffer (pH 4.5) to final concentration of 25 mg/ml. The tail tip blood glucose was measured four times a day, before and 2 hours after feeding in the morning and afternoon. The dose of exogenous insulin treatment for animal was determined according to pre-prandial blood glucose.

Immunosuppression strategy

The immunosuppression treatment was started 2 days before transplantation (day -2). Sirolimus (0.5 mg, q.d., p.o.) and mycophenolate mofetil dispersible tablet (62.5 mg, b.i.d., p.o.) were administered daily since day -2. Diclofenac sodium suppository (50

mg, p.r.) was used 30 minutes before transplantation. ATG (12.5 mg, i.v.) was injected 1 hour before and 48 hours after transplantation. Etanercept (25 mg, i.h.) was used 1 hour before transplantation and days 3 and 7 after transplantation.

Transplant surgeries

The animal was fasted for at least 4 hours and anaesthetized with intramuscular injection of Zoletil®50 at 3-5 mg/kg. Heart rate, temperature, blood oxygenation and blood pressure were monitored in real time during the surgical procedure. E-islets (6000 or 30000 IEQ) were transplanted through B-ultrasound-mediated percutaneous hepatic portal vein injection. Antibiotic treatment was continued for 7 days post transplantation. Two diabetic monkeys were transplanted with 6000 (Monkey 1) or 30000 (Monkey 2) E-islets, respectively. Monkey 1 was used to test the feasibility of hepatic portal injection of E-islets without DSA, while Monkey 2 was used for evaluating the short-term safety and effectiveness of E-islets.

Transplantation of E-islets into diabetic humanized mice

Generation of humanized mice by engraftment of human PBMCs

PBMCs of the patient or an unrelated volunteer were isolated from whole blood using Ficoll gradient, respectively. NCG-hIL15 female mice (6 weeks) were treated with 250 cGy of radiation 4 hours before PBMC infusion. Five million PBMCs were injected into lateral tail vein for each mouse. Efficiency of PBMC engraftment was evaluated by flow cytometry weekly following the injection, by proportion of mCD45/hCD45 cells in mice blood. The percentage of hCD45 cells increased to > 40% within two weeks.

Induction of diabetes, transplantation and evaluation

After identification of engraftment of human PBMCs, humanized mice were treated with 170 mg/kg STZ (Sigma-Aldrich, S0130) by intraperitoneal injection after 4 hours of starvation. One thousand E-islets generated from patient’s EnSCs were transplanted

under the left kidney capsule of 1) NCG-hIL15 mice humanized with the patient's PBMCs, or 2) NCG-hIL15 mice humanized with PBMCs from an unrelated volunteer. Fasting blood glucose were measured every two days. Glucose-stimulated human C-peptide secretion was performed as described above. Animals were sacrificed at 28 days posttransplantation and were examined for graft survival by immunofluorescence of islet markers (C-peptide, Glucagon, PDX1, NKX6-1) in tissue sections.

Clinical studies

Dosage Design

The rationale behind the dosage of 1.2 million IEQ units for E-islet transplantation of this T2D patient is based on the following facts: 1) there are approximately 4~6 million IEQs of islets in a healthy person, and it is estimated that only 1/3 of the islets function upon glucose stimulation under physiological condition, which means ~1.5 million IEQs of islets might be enough for glycemic control. This is confirmed by the clinical observation that transplantation of 800,000 IEQs of cadaveric islets would normally lead to independency of exogenous insulin in most T1D patients (see references below); 2) the patient's endogenous beta cell mass was significantly diminished (estimated at 50% reduction at least), judging from the pre and the post-prandial c-peptide levels; and 3) a significant number of E-islets are likely lost during the vascularization process, as E-islets contain only pancreatic endodermal cells but not vascular endothelial cells that are important for the post-transplantation revascularization of cadaveric islets. We chose the dosage of 1.2 million IEQs, as we speculated that 50% of E-islets might be lost during the engraftment, and that the residual 0.6 million IEQs might be enough to supplement the endogenous islet function.

E-islet transplantation

Conforming to the regulatory guidance from the clinical islet transplantation registration (CITR), the patient had an image-guided percutaneous transhepatic islet infusion with a local anesthetic, into the main portal circulation with heparinization.

Patency of the main portal vein was assessed by monitoring portal pressure during infusion of islets, and doppler ultrasonography after the infusion. A total of 1.2 million IEQs of E-islets that were generated as a single batch and passed the release criteria were directly delivered without prior cryopreservation.

Mixed Meal Tolerance Test (MMTT)

After overnight (≥10 hours) fasting, at 7:30 AM, the patient was asked to consume within 5 minutes a standard mixed meal including 200 g steamed buns and 50 mL water at a constant speed. Blood samples were collected before (0 min) and at 15, 30, 60, 120, 180 and 240 minutes after ingesting. Degludec was not administered 24 hours before MMTT, and oral antidiabetic medications were not administrated 20 hours prior to MMTT.

References

Shaprio A M. Islet transplantation in type 1 diabetes: ongoing challenges, refined procedures, and long-term outcome. Rev Diabet Stud, 2012, 9(4): 385-406.

Shaprio A M, Pokrywczynska M, Ricordi C. Clinical pancreatic islet transplantation. Nat Rev Endocrinol, 2017, 13(5): 268-77.

Shaprio A M, Lakey J R, Ryan E A, et al. Islet transplantation in seven patients with type 1 diabetes mellitus using a glucocorticoid-free immunosuppressive regimen. N Engl J Med, 2000, 343(4): 230-8.

Figure S1

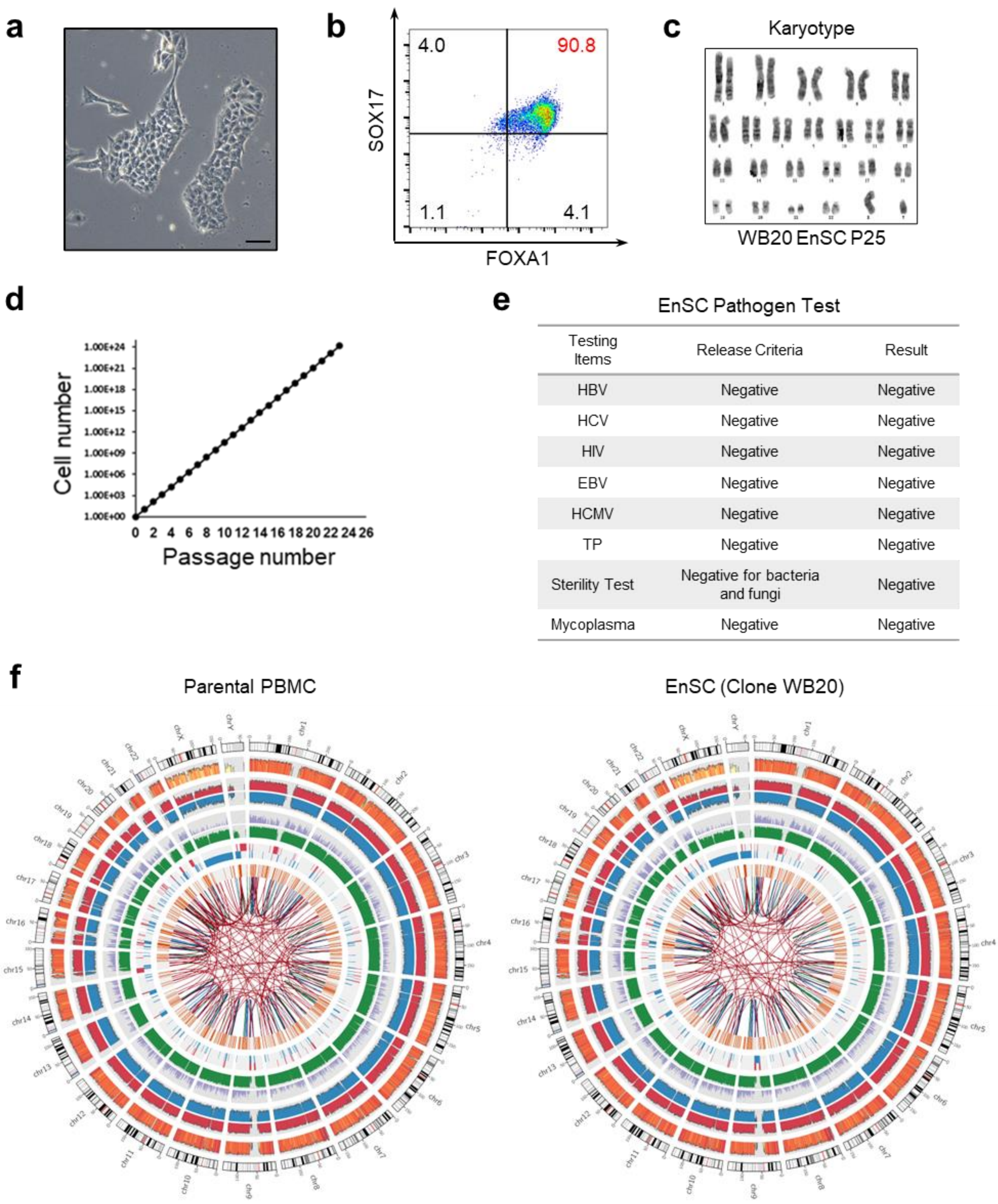

Testing Items	Release Criteria	Result
HBV	Negative	Negative
HCV	Negative	Negative
HIV	Negative	Negative
EBV	Negative	Negative
HCMV	Negative	Negative
TP	Negative	Negative
Sterility Test	Negative for bacteria and fungi	Negative
Mycoplasma	Negative	Negative

From outside to inside: Circle 1: chromosome; circle 2: density map of SNV; circle 3: density map of Indel insertion; circle 4: density map of Indel deletion; circle 5: density map of mutation sites occurring in the coding region; circle 6: density map of mutation sites occurring in the non-coding region; circle 7: location map of CNV; circle 8: location map of SV; circle 9: type association map of SV.

Supplementary Fig. S1. Quality control of EnSCs

a Morphology of EnSCs. **b** Result of FACS, revealing the proportion of SOX17+ / FOXA1+ cells in EnSCs. **c** Karyotyping result of WB20 EnSC line. **d** Growth curve of

EnSCs. **e** Release criteria and the results of pathogen testing for EnSCs. **f** Circos plots of parental PBMC (left) and EnSC (clone WB20) (right) for genomic variations from WGS analysis. From outside to inside, each of nine circles represents one aspect of genomic variations. Circle 1: chromosome; circle 2: density map of SNV (single nucleotide variation); circle 3: density map of Indel insertion; circle 4: density map of Indel deletion; circle 5: density map of mutation sites occurring in the coding region; circle 6: density map of mutation sites occurring in the non-coding region; circle 7: location map of CNV (copy number variation), with red and blue columns indicating copy number gain and loss, respectively; circle 8: location map of SV (structural variation), with orange and green columns indicating deletion and insertion, respectively; circle 9: type association map of SV, with blue, red and green lines indicating inversion, interchromosomal translocation and intrachromosomal translocation, respectively.

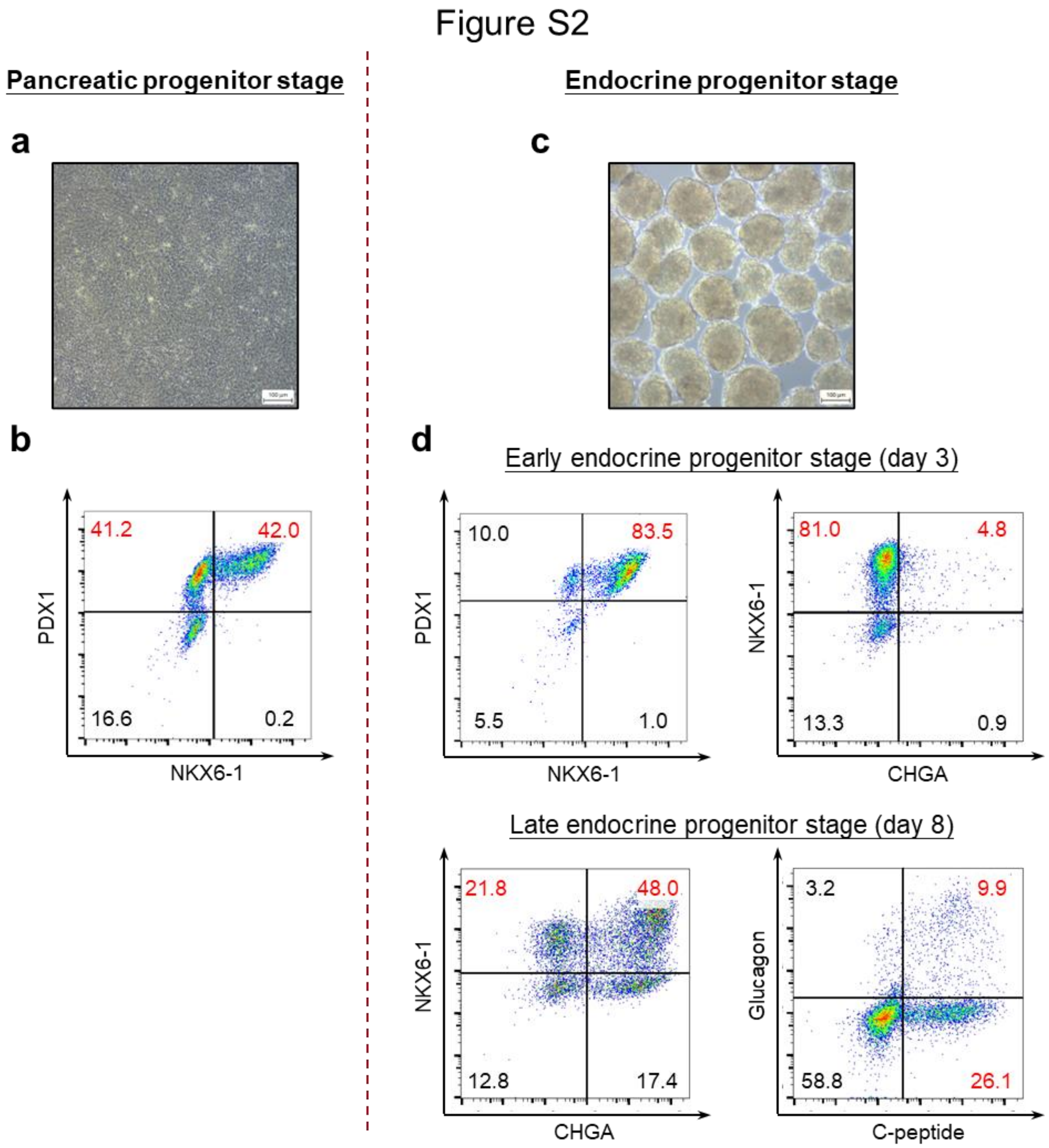

Supplementary Fig. S2. Quality control of intermediate pancreatic differentiation stages of EnSCs

a and **b** Morphology and the cell composition of the differentiation culture at the pancreatic progenitor (PP) stage. **a** is a representative phase contrast image of EnSC-derived PP cells. **b** shows the FACS data revealing the proportion of NKX6-1+ / PDX1+ PP cells at this stage. **c** and **d** Morphology and the cell composition of the differentiation culture at the endocrine progenitor (EP) stage. **c** is a representative phase contrast image of EnSC-derived EP cells. **d** shows the FACS data revealing the proportion of NKX6-1+ / PDX1+ and CHGA- / NKX6-1+ PP cells that are differentiating towards the

CHGA+ endocrine progenitors at early endocrine progenitor stage (day 3 of EP stage) and the proportion of CHGA+ endocrine progenitors and emerging endocrine (C-peptide + or Glucagon+) cells at late endocrine progenitor stage (day 8 of EP stage). Scale bars, 100 μm.

Figure S3

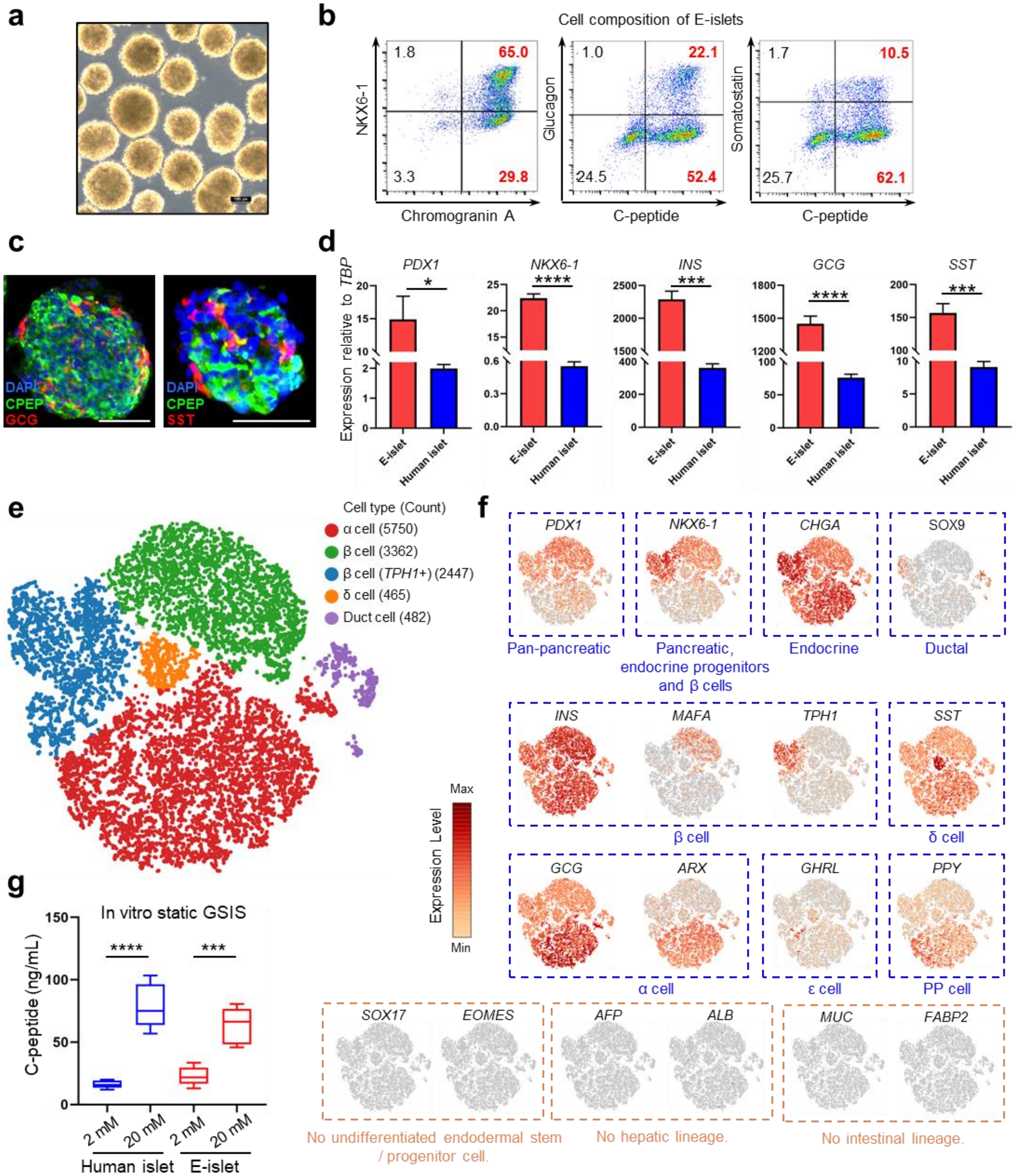

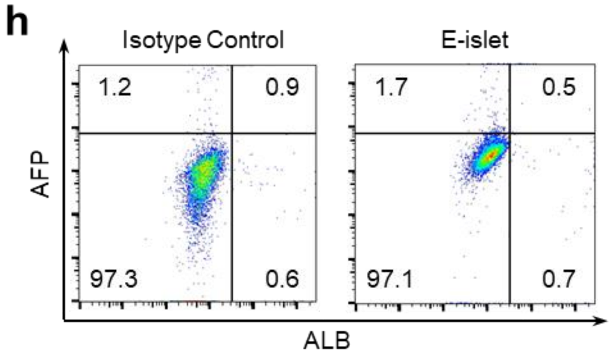

i

E-islet Pathogen Test

Testing Items	Release Criteria	Result
HBV	Negative	Negative
HCV	Negative	Negative
HIV	Negative	Negative
EBV	Negative	Negative
HCMV	Negative	Negative
TP	Negative	Negative
Sterility Test	Negative for bacteria and fungi	Negative
Mycoplasma	Negative	Negative
Endotoxin	< 1 EU/mL	< 0.01 EU/mL

Supplementary Fig. S3. Quality control of E-islets

a Morphology of E-islets (scale bar, 100 μm). **b** Results from FACS analysis of pancreatic endocrine cells in E-islets, with Chromogranin A+ population representing the pan-endocrine compartment, C-peptide+ Glucagon- population representing β cells, Glucagon+ population representing α cells and Somatostatin+ population representing δ cells. **c** immunofluorescence staining of E-islets that have C-peptide (CPEP) positive β cells, glucagon (GCG) positive α cells, as well as somatostatin (SST) positive δ cells (scale bars, 50 μm). **d** Expression levels of the indicated genes in E-islets and adult human islets measured by quantitative RT-PCR (qRT-PCR), among which *PDX1*, *NKX6.1* and Insulin (*INS*) are expressed by adult β cells, while Glucagon (*GCG*) and Somatostatin (*SST*) are typically expressed by adult α cells and δ cells, respectively. **e** and **f** Clustering and gene expression among the subpopulations of E-islets, revealed by single cell transcriptomic analysis (scRNA seq, 10× Genomics). **e** is the tSNE clustering data of scRNA seq, showing the various subpopulations of E-islets (2721 cells with low UMI are excluded from 15244 sequenced cells). **f** shows the expression of representative genes for the cell types (indicated by the boxes with blue dotted lines) in E-islets. **g** C-peptide secretions from human (primary) islets and E-islets in response to low and high glucose stimulations under static conditions (GSIS). **h** FACS data, revealing the nontarget hepatic lineages (Alpha fetoprotein/AFP+ or Albumin/ALB+ cells) are undetectable in E-islets. **i** Release criteria and results of pathogen testing for E-islets.

Figure S4

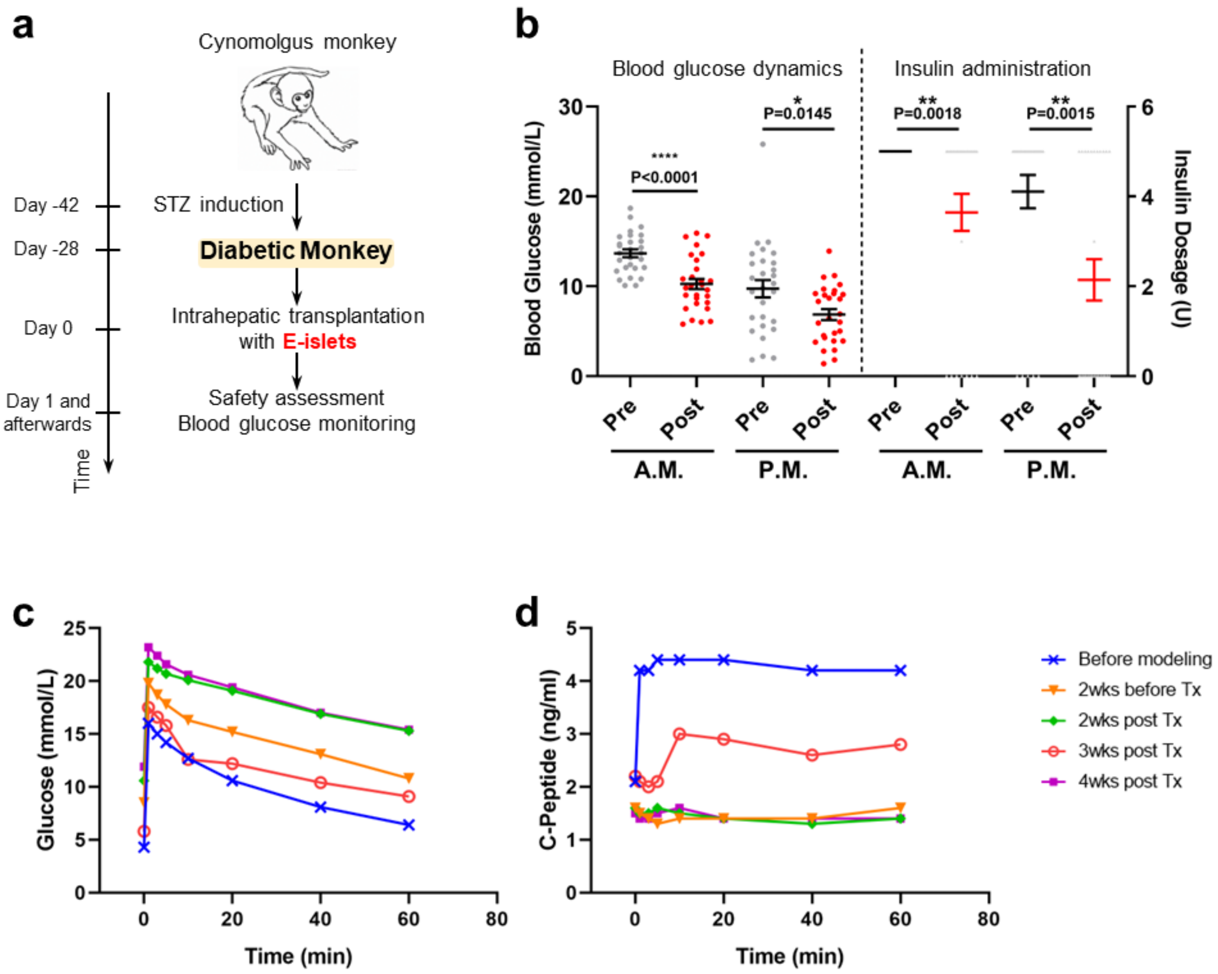

Supplementary Fig. S4. E-islets ameliorate diabetes in STZ-induced diabetic monkeys

a Schematic illustration of the hepatic portal implantation of E-islets into STZ-induced diabetic monkeys. Two diabetic monkeys were transplanted with 6000 (Monkey 1) or 30000 (Monkey 2) E-islets, respectively. Monkey 1 was used to test the feasibility of hepatic portal injection of E-islets without DSA, while Monkey 2 was used for evaluating the short-term safety and effectiveness of E-islets. The tail tip blood glucose was measured before feeding in the morning and afternoon. The dose of exogenous insulin treatment for animal was determined according to preprandial blood glucose. **b** Daily preprandial blood glucose levels (left) and insulin administration dosage (right) during the 28 day-periods before and after surgery (Pre: pre-surgery; Post: post-surgery; A.M.: before breakfast; P.M.: before dinner.). **c** and **d** Results of the 8-time-point intravenous glucose tolerance tests (IVGTT) of the E-islet-transplanted STZ-induced

diabetic monkey. The patterns of blood glucose (**c**) and human C-peptide (**d**) were monitored by IVGTT before diabetes modeling / STZ treatments (before diabetes modeling), 2 weeks before transplantation (2wks before Tx), as well as 2, 3 and 4 weeks posttransplantation (2wks, 3wks, and 4wks post Tx).

Figure S5

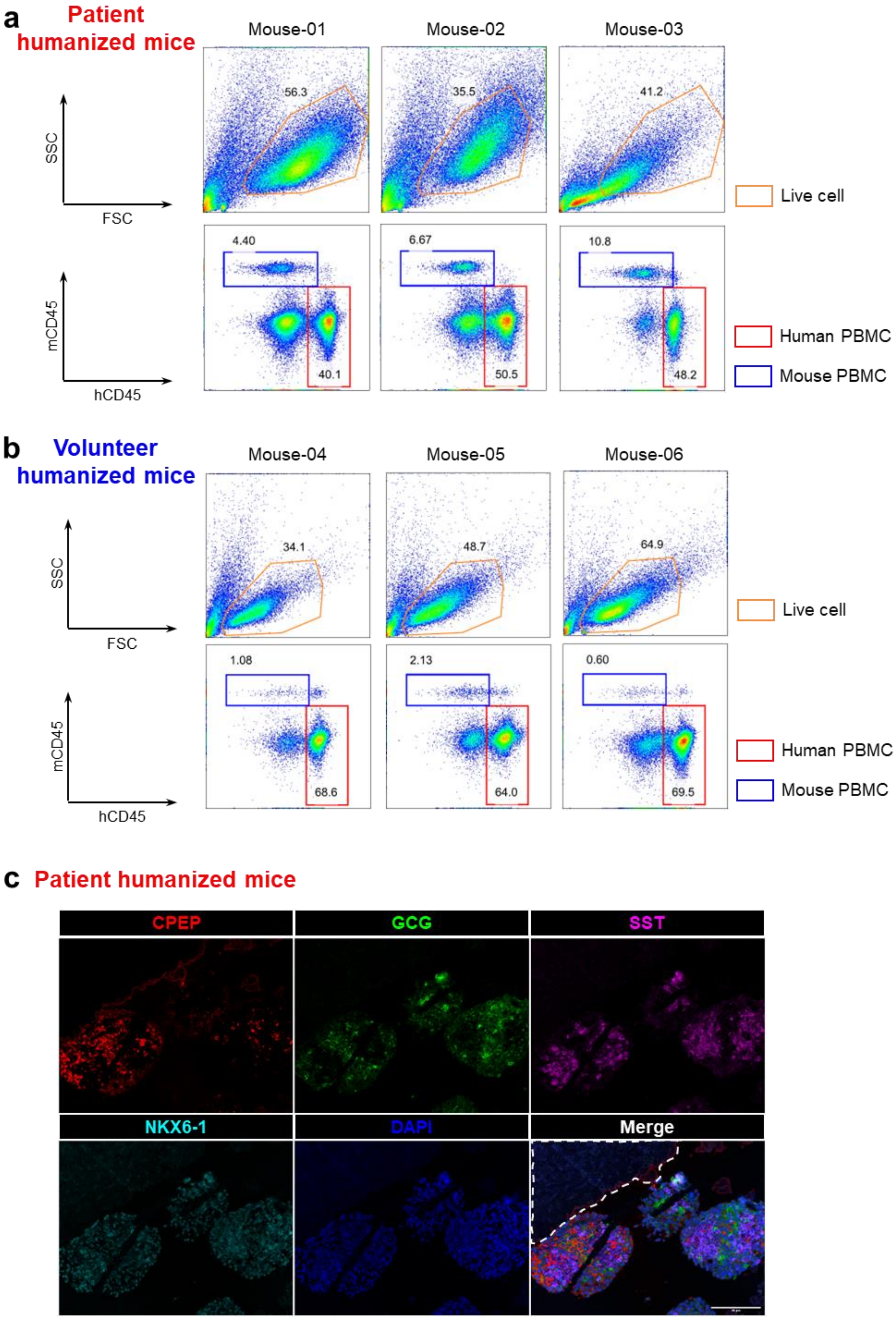
a
Patient humanized mice
Mouse-01
Mouse-02
Mouse-03
SSC
FSC
56.3
35.5
41.2
Live cell
mCD45
hCD45
4.40
40.1
6.67
50.5
10.8
48.2
Human PBMC
Mouse PBMC
b
Volunteer humanized mice
Mouse-04
Mouse-05
Mouse-06
34.1
48.7
64.9
1.08
68.6
2.13
64.0
0.60
69.5
c
Patient humanized mice
CPEP
GCG
SST
NKX6-1
DAPI
Merge

Supplementary Fig. S5. Characterization of humanized mice

a and **b** Characterization of NCG-hIL15 mice humanized with either patient (**a**) or volunteer (**b**)-derived PBMCs by the presence of hCD45+ human cells in peripheral blood. **a** Proportions of live cells (by SSC and FSC), human-derived blood cells (hCD45+) and mouse blood cells (mCD45+), among the three patient humanized mice. **b** Proportions of live cells, human-derived and mouse blood cells, among the three volunteer humanized mice. **c** Immunofluorescence staining of the grafts harvested under kidney capsule of the patient humanized mice for the presence of human β cells (C-peptide + and NKX6-1 +), α cells (Glucagon +), and δ cells (Somatostatin +); C-peptide (CPEP, red), Glucagon (GCG, green), Somatostatin (SST, violet) and NKX6-1 (Cyan); the area defined by the white dotted line indicating the mouse kidney tissue; scale bar, 50 μm.

Figure S6

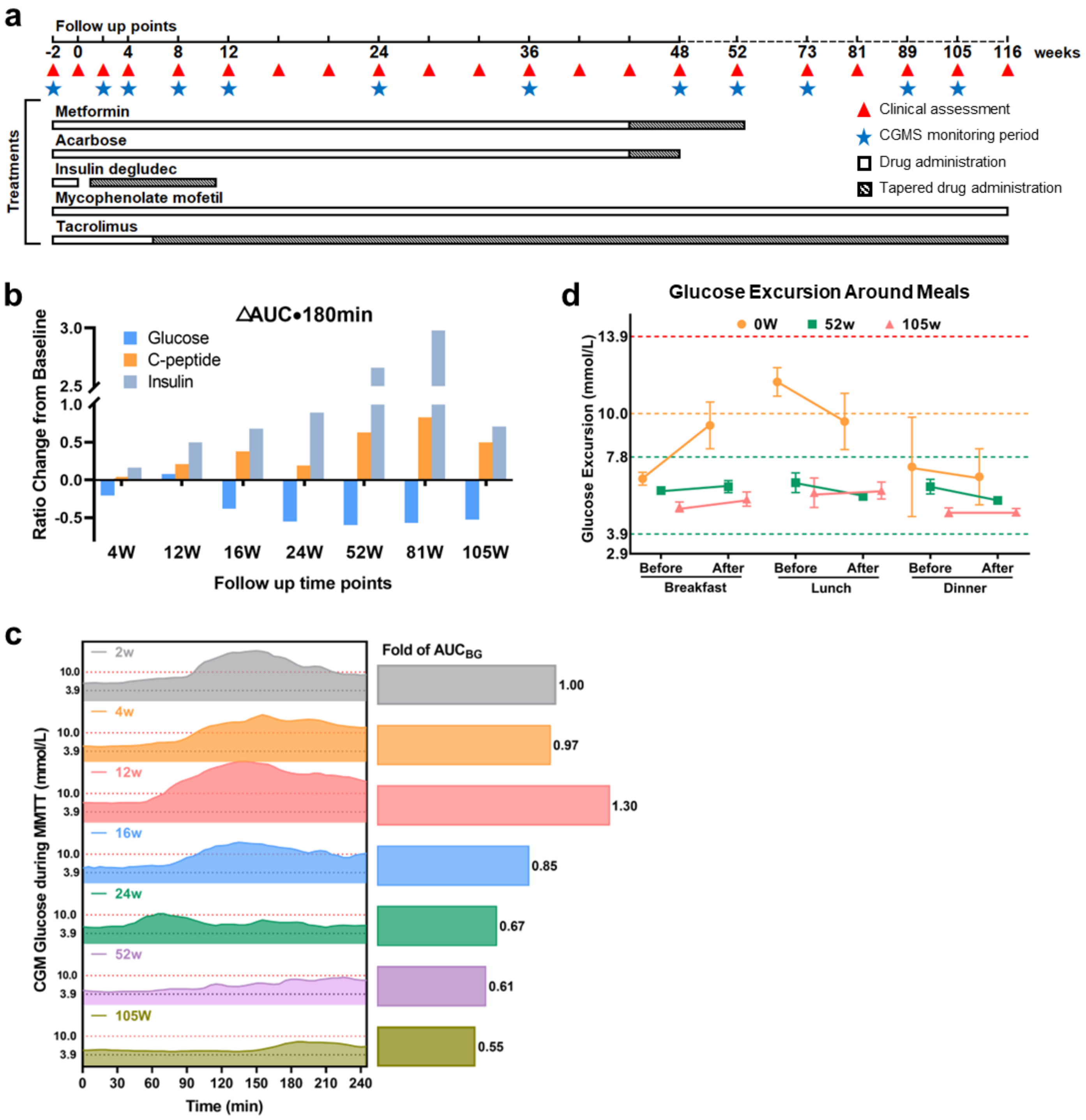

Supplementary Fig. S6. Clinical assessments and outcomes of glycemic control

a Schematic illustration of follow-up time points for routine and disease-specific clinical assessments, as well as the treatments the patient received during the whole follow-up period. Examinations of endocrine function and diabetes–specific parameters by mixed-meal tolerance tests (MMTT) were performed at baseline and at 4, 8, 12, 16,

20, 24, 36 and 48 weeks and thereafter at specified time points. The glycemic control of the patient was measured with a 24-hour real-time blood glucose monitoring (CGM) system. The baseline and follow-up CGM glucose values were measured throughout the first 52 weeks and thereafter during specified time periods, and the mean duration of CGM device wearing is at least 3 days. The main preconditioning regimen included antihyperglycemic medication and immunosuppressant treatments. The antidiabetic treatment included metformin (0.75 g bid, tapered from week 44 and interrupted from week 56) and acarbose (50 mg tid, tapered from the week 44 and interrupted from week 48). The insulin analog degludec had been administrated since 2021 (20 U once daily at bedtime) but interrupted right after E-islet transplantation, and had been resumed and tapered from week 2, and was stopped at the end of week 11. Graft-versus-host disease was treated with mycophenolate mofetil (administered since kidney transplantation at 0.5 g bid) and tacrolimus (administered orally after kidney transplantation at 1~3 mg bid, depending on the serum FK506 concentrations). ☆ represents the follow-up time points of clinical assessment, and △ represents the CGMS monitoring periods. The checkerboard design represents the tapering period. **b** Areas under the curves (AUCs) derived from the 5-point (0, 30, 60, 120, 180 min) intravenous glucose (Figure **1j**), C-peptide (Figure **1k**) and insulin (Figure **1l**) values in the results of the mixed meal tolerance tests (MMTT). **c** Results of continuous glucose monitoring during MMTTs (0-240 minutes) at various follow-up time points, measured by CGM device at intervals of every 5 minutes. The horizontal dotted lines at 3.9 and 10.0 mM demarcate the target glucose range. Fold of AUCs (right panel) are the areas under the curves of each follow-up time points, normalized to the AUC of week 2 (2W). **d** Prandial glucose excursions (mean amplitude glucose excursion, MAGE), the gold standard of blood glucose variability, represented by mean glucose values (within 95% ranges) of 1.5 hours before and 2 hours after each meal at baseline, week 52 and week 105. The green horizontal lines at 3.9 and 7.8 mM demarcate the target glucose excursion for healthy individuals.

Figure S7

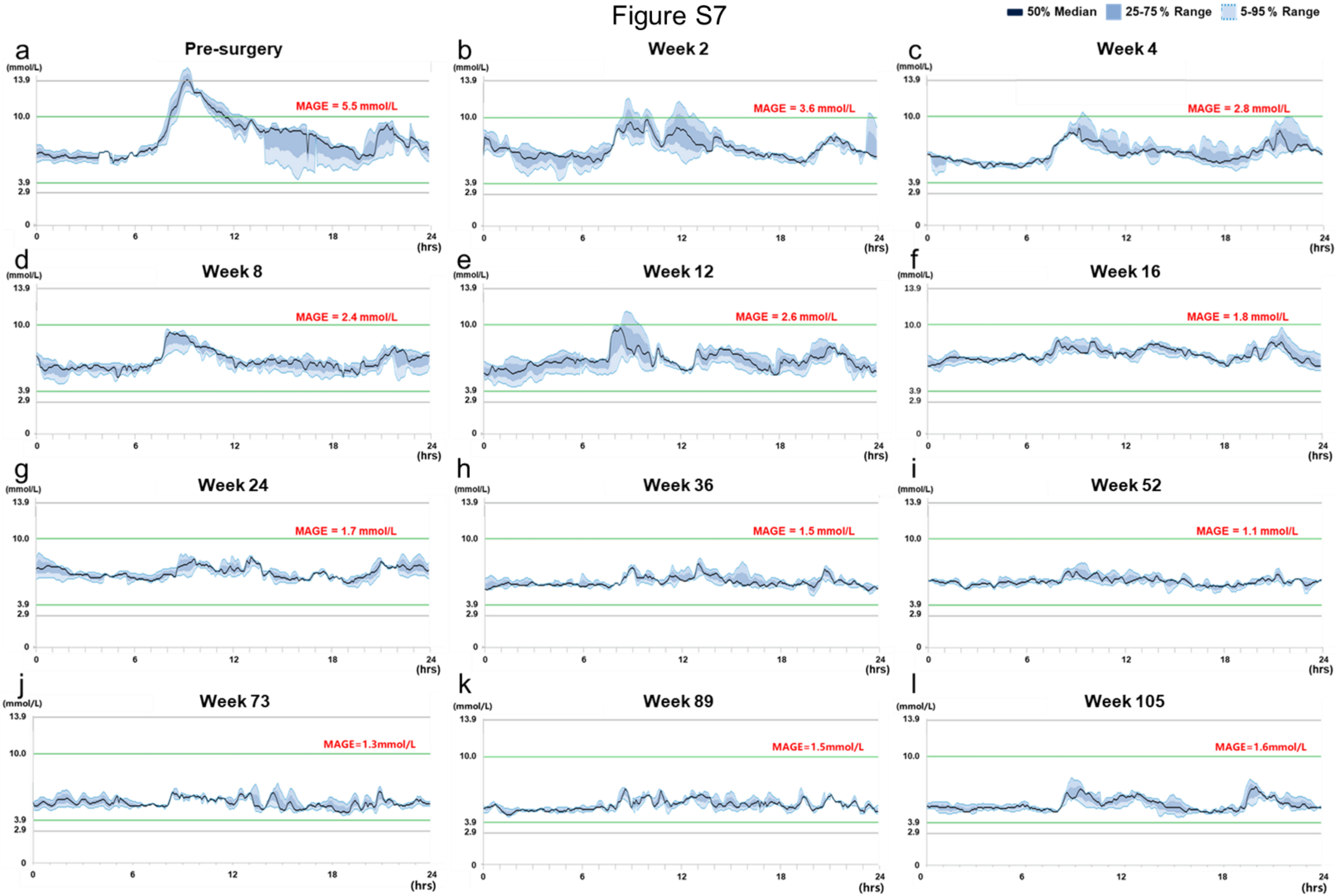
50% Median
25-75 % Range
5-95 % Range
a Pre-surgery
MAGE = 5.5 mmol/L
b Week 2
MAGE = 3.6 mmol/L
c Week 4
MAGE = 2.8 mmol/L
d Week 8
MAGE = 2.4 mmol/L
e Week 12
MAGE = 2.6 mmol/L
f Week 16
MAGE = 1.8 mmol/L
g Week 24
MAGE = 1.7 mmol/L
h Week 36
MAGE = 1.5 mmol/L
i Week 52
MAGE = 1.1 mmol/L
j Week 73
MAGE=1.3mmol/L
k Week 89
MAGE=1.5mmol/L
l Week 105
MAGE=1.6mmol/L
(mmol/L)
13.9
10.0
3.9
2.9
0
0
6
12
18
24
(hrs)

Supplementary Fig. S7. Continuous glucose monitoring at various follow-up time points

a-l Continuous glucose monitoring (CGM) traces at presurgery (**a**) and various follow-up time points (**b-l**). For each follow-up time point, GCM data were collected from continuous 72 hours. The median (navy lines) the 25-75% (medium blue areas) and 5-95% (pale blue areas) ranges are shown, with the green horizontal lines demarcating the target glucose range (3.9 to 10.0 mM) for diabetic patients. The prandial glucose excursions (MAGE), calculated from mean glucose values (within 95% ranges), are listed on each panel in red.

Supplementary Table Information

Supplementary Table S1. Exploratory objectives

Tab.S1: Exploratory objectives

Parameter	Baseline	2W	4W	8W	12W	16W	24W	36W	52W	85W	113W	Normal Range
**HbA1c concentration (%)**	6.6	-	6.8	7	7	-	6.5	6.2	5.2	5.5	4.6	4.0-6.0
Change from baseline (%)	0.00	-	0.20	0.40	0.40	-	-0.10	-0.40	-1.40	-1.10	-2.00	

CGMS values	Baseline	2W	4W	8W	12W	16W	24W	36W	52W	73W	89W	105W
**Average [(Mean ± SD) mM]**	7.77 ± 2.04	7.18 ± 1.41	6.69 ± 1.13	6.56 ± 1.02	6.65 ± 1.2	7.24 ± 0.71	6.87 ± 0.71	6.06 ± 0.57	6.08 ± 0.48	5.50 ± 0.59	5.52 ± 0.62	5.56 ± 0.69
**MAGE (mM)**	5.50	3.60	2.80	2.40	2.60	1.80	1.70	1.50	1.10	1.30	1.50	1.60
**Proportion of TITR (3.9-7.8mM, %)**	56.70	77.80	85.40	89.50	89.10	81.10	88.40	99.20	99.60	100.00	100.00	100.00
Change from baseline (%)	-	21.10	28.70	32.80	32.40	24.40	31.70	42.50	42.90	43.30	43.30	43.30
**Proportion of TIR (3.9-10.0mM, %)**	87.70	94.10	98.70	100.00	98.10	100.00	100.00	100.00	100.00	100.00	100.00	100.00
Change from baseline (%)	-	6.40	11.00	12.30	10.40	12.30	12.30	12.30	12.30	12.30	12.30	12.30
**TAR >10.0 mM, (%)**	10.70	5.90	1.30	0.00	1.90	0.00	0.00	0.00	0.00	0.00	0.00	0.00
Change from baseline (%)	-	-4.80	-9.40	-10.70	-8.80	-10.70	-10.70	-10.70	-10.70	-10.70	-10.70	-10.70
**TAR >13.9 mM, (%)**	1.30	0.00	0.00	0.00	0.00	0.00	0.00	0.00	0.00	0.00	0.00	0.00
Change from baseline (%)	-	-1.30	-1.30	-1.30	-1.30	-1.30	-1.30	-1.30	-1.30	-1.30	-1.30	-1.30
**TBR <3.9 mM, (%)**	0.30	0.00	0.00	0.00	0.00	0.00	0.00	0.00	0.00	0.00	0.00	0.00
Change from baseline (%)	-	-0.30	-0.30	-0.30	-0.30	-0.30	-0.30	-0.30	-0.30	-0.30	-0.30	-0.30
**TBR <2.9 mM, (%)**	0.00	0.00	0.00	0.00	0.00	0.00	0.00	0.00	0.00	0.00	0.00	0.00
**Incidence of episode of hypoglycaemia (≤70 mg/dL), n per day**	0.30	0.00	0.00	0.00	0.00	0.00	0.00	0.00	0.00	0.00	0.00	0.00
**AUC_{MMTT}**	-	2673.75	2600.50	3294.75	3467.25	2272.25	1800.00	1809.75	1638.25	N/A	N/A	1474.00
Ratio change from 2 week	-	1.00	0.97	1.23	1.30	0.85	0.67	0.68	0.61	N/A	N/A	0.55

HbA1c=glycated haemoglobin A1c.
Data are estimated mean (SE); TAR = time above range; TBR = time below range; TIR = time in range; TITR = Time in tight range.
Time (minutes), Percent (%), N/A: Not available.

Supplementary Table S2. Summary of SNV discovered from whole genome sequencing of EnSCs compared to parental PBMCs

#CHROM	Start	End	REF	ALT	QUAL	Func.refGene	Gene.refGene	GeneDetail.refGene	ExonicFunc.refGene	AAChange.refGene	WB20	WB_PBMC
chr1	1081649	1081649	C	G	1414.3	downstream	C1orf159	.	.	.	0/1	1/1
chr1	1363602	1363602	T	C	1032.05	upstream	MXRA8	.	.	.	1/1	0/1
chr1	6104619	6104619	C	G	1244.53	UTR3	CHD5	NM_015557.3:c.*855G>C	.	.	0/1	1/1
chr1	10358685	10358685	A	G	795.18	ncRNA_exonic	LOC105376725	.	.	.	0/1	1/1
chr1	11777648	11777648	A	G	3523.54	ncRNA_exonic	C1orf167-AS1	.	.	.	0/1	1/1
chr1	16617656	16617656	T	C	9999.19	ncRNA_exonic	LOC105376794	.	.	.	1/1	0/1
chr1	16620359	16620359	A	G	9634.16	ncRNA_exonic	LOC105376794	.	.	.	0/1	1/1
chr1	16623287	16623287	T	C	144.16	ncRNA_exonic	LOC105376794	.	.	.	0/1	0/0
chr1	16727673	16727673	G	A	201.27	downstream	TRG-CCC5-1	.	.	.	0/1	0/0
chr1	21938117	21938117	T	G	1856.13	upstream	HSPG2	.	.	.	1/1	0/1
chr1	24744706	24744706	A	T	946.48	upstream	CLIC4	.	.	.	1/1	0/1
chr1	25819427	25819427	T	A	1170.35	upstream;downstream	MTFR1L;LOC646471	.	.	.	0/1	1/1
chr1	30732548	30732548	C	T	2235.13	UTR3	LAPTM5	NM_006762.3:c.*1280G>A,XM_011542098.2:c.*1280G>A	.	.	1/1	0/1
chr1	30732949	30732949	T	C	3108.13	UTR3	LAPTM5	NM_006762.3:c.*879A>G,XM_011542098.2:c.*879A>G	.	.	1/1	0/1
chr1	32280150	32280150	G	A	386.13	exonic	LCK	.	nonsynonymous_SNV	LCK:XM_011541453.3:exon10:c.G1288A:p.V430M,LCK:NM_001330468.2:exon11:c.G1114A:p.V372M,LCK:XM_024447046.1:exon11:c.G1441A:p.V481M,LCK:XM_024447047.1:exon11:c.G1441A:p.V481M,LCK:NM_001042771.2:exon12:c.G1267A:p.V423M,LCK:NM_005356.5:exon12:c.G1267A:p.V423M	0/1	0/0
chr1	52140816	52140816	G	C	1648.13	upstream	LOC107984956	.	.	.	1/1	0/1
chr1	70132486	70132486	G	T	83.93	UTR3	LRRC7	NM_001366838.3:c.*10599G>T,NM_001370785.2:c.*10599G>T,NM_001366839.3:c.*10599G>T,NM_001330635.3:c.*10599G>T,NM_001366841.1:c.*10753G>T,NM_001350216.2:c.*10753G>T	.	.	0/1	0/0
chr1	85277901	85277901	C	A	761.16	ncRNA_exonic	LOC646626	.	.	.	1/1	0/1
chr1	85491071	85491071	C	A	73.93	ncRNA_exonic	LOC107985054	.	.	.	0/1	0/0
chr1	85629715	85629715	C	A	1283.2	ncRNA_exonic	LOC107985057,LOC112268231	.	.	.	0/1	1/1
chr1	94541903	94541903	G	A	52.14	upstream	F3	.	.	.	0/1	0/0
chr1	109503471	109503471	G	A	94.14	downstream	AMIGO1	.	.	.	0/1	0/0
chr1	145992806	145992806	T	A	4579.13	UTR3	TXNIP	NM_001313972.2:c.*1045A>T,XM_017000085.2:c.*1162A>T,NM_006472.6:c.*1045A>T	.	.	1/1	0/1
chr1	145992816	145992816	G	C	4654.13	UTR3	TXNIP	NM_001313972.2:c.*1035C>G,XM_017000085.2:c.*1152C>G,NM_006472.6:c.*1035C>G	.	.	1/1	0/1
chr1	145993449	145993449	G	A	2444.13	UTR3	TXNIP	NM_001313972.2:c.*402C>T,XM_017000085.2:c.*519C>T,NM_006472.6:c.*402C>T	.	.	1/1	0/1
chr1	148158811	148158811	C	A	1198.57	downstream	LOC105371227	.	.	.	1/1	0/1
chr1	155334450	155334450	G	A	870.14	downstream	ASH1L	.	.	.	1/1	0/1
chr1	155334451	155334451	A	G	533.93	downstream	ASH1L	.	.	.	1/1	0/1
chr1	156929638	156929638	A	C	71.13	exonic	LRRC71	.	nonsynonymous_SNV	LRRC71:XM_011509239.2:exon7:c.A591C:p.E197D,LRRC71:XM_011509240.3:exon7:c.A504C:p.E168D,LRRC71:NM_144702.3:exon11:c.A1149C:p.E383D,LRRC71:XM_005244926.3:exon11:c.A1149C:p.E383D,LRRC71:XM_005244927.3:exon11:c.A1149C:p.E383D,LRRC71:XM_005244928.2:exon11:c.A1149C:p.E383D,LRRC71:XM_006711186.3:exon11:c.A1149C:p.E383D,LRRC71:XM_006711187.3:exon11:c.A1149C:p.E383D,LRRC71:XM_017000459.2:exon11:c.A1149C:p.E383D,LRRC71:XM_017000460.1:exon11:c.A504C:p.E168D,LRRC71:XM_024453542.1:exon11:c.A504C:p.E168D,LRRC71:XM_006711185.3:exon12:c.A1068C:p.E356D,LRRC71:XM_017000461.1:exon12:c.A504C:p.E168D,LRRC71:XM_017000462.2:exon12:c.A504C:p.E168D	0/1	0/0
chr1	158100732	158100732	G	C	3049.54	downstream	KIRREL1	.	.	.	0/1	1/1
chr1	160841903	160841903	T	C	285.13	splicing	CD244	NM_001166663.2:exon2:c.62-2A>G,NM_016382.4:exon2:c.62-2A>G,NM_001166664.2:exon2:c.62-2A>G,XM_011509621.2:exon2:c.62-2A>G,XM_011509622.2:exon2:c.62-2A>G	.	.	0/1	0/0
chr1	206587354	206587354	A	T	4053.52	UTR3	RASSF5	NM_182664.4:c.*506A>T,NM_182663.4:c.*376A>T,NM_182665.4:c.*376A>T	.	.	1/1	0/1
chr1	206587362	206587362	C	T	4191.15	UTR3	RASSF5	NM_182664.4:c.*514C>T,NM_182663.4:c.*384C>T,NM_182665.4:c.*384C>T	.	.	1/1	0/1
chr1	210326932	210326932	T	C	1769.16	upstream	HHAT	.	.	.	0/1	1/1
chr1	210326934	210326934	T	C	168.91	upstream	HHAT	.	.	.	0/1	0/0
chr1	220112475	220112475	A	C	54.52	ncRNA_exonic	LOC105373475	.	.	.	0/1	0/0
chr1	220112581	220112581	C	A	47.32	ncRNA_exonic	LOC105373475	.	.	.	0/1	0/0
chr2	6373521	6373521	G	C	158.13	ncRNA_exonic	LINC01247	.	.	.	0/1	0/0
chr2	7058339	7058339	A	G	1053.24	UTR3	RNF144A	XM_017005401.2:c.*12930A>G	.	.	1/1	0/1
chr2	32316038	32316038	A	C	1862.2	UTR3	YIPF4	NM_032312.4:c.*10412A>C	.	.	1/1	0/1
chr2	32316041	32316041	C	A	1897.2	UTR3	YIPF4	NM_032312.4:c.*10415C>A	.	.	1/1	0/1
chr2	47373967	47373967	T	C	2069.18	exonic	EPCAM	.	nonsynonymous_SNV	EPCAM:NM_002354.3:exon3:c.T344C:p.M115T	1/1	0/1
chr2	66425582	66425582	G	A	1899.37	ncRNA_exonic	MEIS1-AS3	.	.	.	1/1	0/1
chr2	74653527	74653527	T	A	165.13	upstream	SEMA4F	.	.	.	0/1	0/0
chr2	95669457	95669457	T	A	43.14	downstream	LOC101926959	.	.	.	0/1	0/0
chr2	95669463	95669463	T	A	46.13	downstream	LOC101926959	.	.	.	0/1	0/0
chr2	152120189	152120189	T	A	2244.13	UTR3	STAM2	NM_005843.6:c.*385A>T	.	.	1/1	0/1
chr2	173074688	173074688	T	C	1273.16	upstream	MAP3K20	.	.	.	1/1	0/1
chr2	173074691	173074691	T	C	1282.16	upstream	MAP3K20	.	.	.	1/1	0/1
chr2	174330091	174330091	G	T	408.13	ncRNA_exonic	LINC01305	.	.	.	0/1	0/0
chr2	175626807	175626807	T	C	281.13	ncRNA_exonic	LOC107985962	.	.	.	0/1	0/0
chr2	236124193	236124193	G	A	471.92	UTR3	AGAP1	NM_014914.5:c.*71G>A,NM_001037131.3:c.*71G>A,XM_006712235.3:c.*71G>A,XM_006712234.3:c.*71G>A,XM_005246059.4:c.*71G>A,XM_011510549.2:c.*71G>A,XM_017003282.1:c.*71G>A,XM_006712239.3:c.*71G>A,XM_011510548.2:c.*71G>A,XM_006712237.3:c.*71G>A,XM_011510547.2:c.*71G>A,XM_024452672.1:c.*71G>A	.	.	0/1	0/0
chr2	237565690	237565690	T	C	626.14	upstream	PRLH	.	.	.	0/1	0/0
chr2	241352963	241352963	G	T	3119.12	UTR3	SEPTIN2	NM_001349306.1:c.*1026G>T,NM_001349315.1:c.*1026G>T,NM_006155.2:c.*1026G>T,NM_001008491.2:c.*1026G>T,XM_024452926.1:c.*1026G>T,XM_017004212.2:c.*1026G>T,XM_024452923.1:c.*1026G>T,NM_001349310.1:c.*1026G>T,NM_001349314.1:c.*1026G>T,NM_001349304.1:c.*1026G>T,NM_001349312.1:c.*1026G>T,NM_001349308.1:c.*1026G>T,NM_001349309.1:c.*1026G>T,NM_001349302.1:c.*1026G>T,NM_001349307.1:c.*1026G>T,NM_001349288.1:c.*1026G>T,NM_001349291.1:c.*1026G>T,NM_001008492.2:c.*1026G>T,NM_001321035.1:c.*1026G>T,NM_001349311.1:c.*1026G>T,NM_001349305.1:c.*1026G>T,NM_001321029.1:c.*1026G>T,NM_001349287.1:c.*1026G>T,NM_001349313.1:c.*1026G>T,NM_001321031.1:c.*1026G>T,N	.	.	1/1	0/1

								M_001282973.1:c.*1026G>T,NM_001282972.1:c.*1026G>T,NM_001321034.1:c.*1026G>T,XM_024452925.1:c.*1026G>T,XM_017004205.2:c.*1050G>T,NM_004404.5:c.*1026G>T,NM_001321032.2:c.*1050G>T,NM_001349289.2:c.*1050G>T,XM_024452922.1:c.*1026G>T,XM_024452924.1:c.*1026G>T,NM_001349290.1:c.*1026G>T,NM_001321030.2:c.*1026G>T,NM_001321033.2:c.*1026G>T,XM_024452920.1:c.*1026G>T,XM_024452921.1:c.*1050G>T				
chr3	5442521	5442521	A	G	200.65	upstream	LOC105376936	.	.	.	0/1	0/0
chr3	38492818	38492818	G	T	71.99	UTR3	ACVR2B	NM_001106.4:c.*9486G>T,XM_017007515.2:c.*9486G>T,XM_017007514.1:c.*9486G>T,XM_005265583.3:c.*9486G>T,XM_017007516.1:c.*9486G>T	.	.	0/1	0/0
chr3	71676745	71676745	C	T	1398.99	UTR3	EIF4E3	NM_001134651.2:c.*7937G>A,NM_001282886.2:c.*7937G>A,NM_173359.5:c.*7937G>A	.	.	1/1	0/1
chr3	113652110	113652110	T	A	1645.74	UTR3	USF3	NM_001009899.4:c.*2834A>T,XM_024453391.1:c.*2834A>T,XM_024453392.1:c.*2834A>T,XM_005247208.4:c.*2834A>T,XM_017005871.1:c.*2834A>T,XM_017005872.1:c.*2834A>T	.	.	1/1	0/1
chr3	121259054	121259054	G	A	233.13	exonic	STXBP5L	.	nonsynonymous_SNV	STXBP5L:XM_011513332.2:exon4:c.G392A:p.R131Q,STXBP5L:NM_001308330.2:exon18:c.G1844A:p.R615Q,STXBP5L:NM_001348343.2:exon18:c.G1844A:p.R615Q,STXBP5L:NM_001348344.2:exon18:c.G1844A:p.R615Q,STXBP5L:NM_001348345.2:exon18:c.G1844A:p.R615Q,STXBP5L:NM_014980.3:exon18:c.G1844A:p.R615Q,STXBP5L:XM_006713825.3:exon18:c.G1844A:p.R615Q,STXBP5L:XM_011513331.2:exon18:c.G1844A:p.R615Q,STXBP5L:XM_011513333.3:exon18:c.G1844A:p.R615Q,STXBP5L:XM_017007534.1:exon18:c.G1844A:p.R615Q,STXBP5L:XM_017007535.2:exon18:c.G1844A:p.R615Q,STXBP5L:XM_024453832.1:exon19:c.G1283A:p.R428Q	0/1	0/0
chr3	122359628	122359628	A	T	1020.57	UTR3	CCDC58	XM_011512410.2:c.*241T>A,NM_001308326.1:c.*241T>A,NM_001017928.4:c.*241T>A	.	.	0/1	1/1
chr3	125995824	125995824	T	A	464.57	upstream	ALG1L	.	.	.	0/1	0/0
chr3	179323100	179323100	T	C	179.14	UTR5	ZNF639	NM_001375803.1:c.-5194T>C,NM_001303426.2:c.-5194T>C,NM_001375800.1:c.-5194T>C,NM_001375804.1:c.-5194T>C,NM_001303425.2:c.-5194T>C	.	.	0/1	0/0
chr3	197671117	197671117	T	A	453.13	UTR3	RUBCN	NM_014687.4:c.*3901A>T,NM_001346873.1:c.*3901A>T,NM_001145642.4:c.*3901A>T,XM_017007543.1:c.*3901A>T,XM_005269374.3:c.*3901A>T,XM_006713828.3:c.*3901A>T,XM_017007544.1:c.*3901A>T,XM_006713827.3:c.*3901A>T,XM_006713830.3:c.*3901A>T,XM_006713829.4:c.*3901A>T,XM_006713831.4:c.*3901A>T,XM_017007545.2:c.*3901A>T,XM_024453838.1:c.*3901A>T	.	.	0/1	0/0
chr4	1026311	1026311	G	A	71.27	UTR3	FGFRL1	XM_024454092.1:c.*964G>A,XM_024454093.1:c.*964G>A,NM_001004356.3:c.*964G>A,NM_001004358.1:c.*964G>A,NM_001370296.1:c.*964G>A,NM_021923.3:c.*964G>A	.	.	0/1	0/0
chr4	17486910	17486910	C	T	4255.52	UTR3	QDPR	NM_001306140.1:c.*221G>A,NM_000320.3:c.*221G>A	.	.	1/1	0/1
chr4	17486911	17486911	A	G	4255.52	UTR3	QDPR	NM_001306140.1:c.*220T>C,NM_000320.3:c.*220T>C	.	.	1/1	0/1
chr4	18024109	18024109	A	T	1677.22	upstream	LCORL	.	.	.	0/1	1/1
chr4	53058318	53058318	A	G	286.13	UTR3	SCFD2	XM_011534375.3:c.*3492T>C	.	.	0/1	0/0
chr4	55636140	55636140	G	T	2474.13	exonic	NMU	.	nonsynonymous_SNV	NMU:NM_001292045.2:exon1:c.C53A:p.A18E,NMU:NM_001292046.2:exon1:c.C53A:p.A18E,NMU:NM_006681.4:exon1:c.C53A:p.A18E	1/1	0/1
chr4	72999279	72999279	C	T	3305.52	downstream	LOC105377273	.	.	.	0/1	1/1
chr4	73098099	73098099	T	G	308.13	exonic	ANKRD17	.	nonsynonymous_SNV	ANKRD17:NM_198889.3:exon25:c.A4242C:p.K1414N,ANKRD17:XM_005265671.4:exon25:c.A4239C:p.K1413N,ANKRD17:XM_017008012.1:exon25:c.A3903C:p.K1301N,ANKRD17:XM_017008013.1:exon25:c.A3900C:p.K1300N,ANKRD17:NM_001286771.3:exon26:c.A4656C:p.K1552N,ANKRD17:NM_015574.2:exon26:c.A4992C:p.K1664N,ANKRD17:NM_032217.5:exon26:c.A4995C:p.K1665N,ANKRD17:XM_017008011.1:exon26:c.A4653C:p.K1551N	0/1	0/0
chr4	81030757	81030757	G	A	2373.13	UTR5	BMP3	NM_001201.5:c.-528G>A	.	.	0/1	1/1
chr4	158872752	158872752	A	T	1409.52	UTR3	FNIP2	XM_005263160.3:c.*1254A>T	.	.	1/1	0/1
chr4	189124355	189124355	C	T	1275.77	ncRNA_exonic	LOC105377613	.	.	.	1/1	0/1
chr5	784861	784861	T	C	294.14	ncRNA_exonic	LOC101928768	.	.	.	0/1	0/0
chr5	784870	784870	A	T	294.14	ncRNA_exonic	LOC101928768	.	.	.	0/1	0/0
chr5	1929527	1929527	A	G	2229.55	upstream	LOC112267929	.	.	.	0/1	1/1
chr5	1929577	1929577	C	A	1558.68	upstream	LOC112267929	.	.	.	0/1	1/1
chr5	39075320	39075320	C	T	1640.16	upstream	RICTOR	.	.	.	1/1	0/1
chr5	61305252	61305252	G	T	640.57	downstream	LOC105378993	.	.	.	0/1	1/1
chr5	171309341	171309341	A	C	55.79	UTR5	TLX3	NM_021025.4:c.-25A>C	.	.	0/1	0/0
chr5	171309342	171309342	G	C	57.61	UTR5	TLX3	NM_021025.4:c.-24G>C	.	.	0/1	0/0
chr6	1707631	1707631	C	T	456.14	ncRNA_exonic	LOC107986513	.	.	.	0/1	0/0
chr6	16327678	16327678	A	C	2389.13	exonic	ATXN1	.	nonsynonymous_SNV	ATXN1:NM_001128164.2:exon7:c.T633G:p.H211Q,ATXN1:NM_000332.3:exon8:c.T633G:p.H211Q	1/1	0/1
chr6	17614642	17614642	G	T	1492.16	downstream	NUP153	.	.	.	1/1	0/1
chr6	27702359	27702359	A	T	430.13	ncRNA_exonic	LINC01012	.	.	.	0/1	0/0
chr6	33889787	33889787	C	T	2561.57	ncRNA_exonic	LINC01016	.	.	.	1/1	0/1
chr6	36232910	36232910	C	T	1144.83	downstream	BRPF3	.	.	.	1/1	0/1
chr6	36602589	36602589	T	C	2653.13	UTR3	SRSF3	NM_003017.5:c.*600T>C	.	.	1/1	0/1
chr6	38171886	38171886	T	A	34.93	UTR3	BTBD9	NM_001172418.1:c.*3099A>T,NM_152733.2:c.*3099A>T,NM_052893.2:c.*3099A>T,NM_001099272.2:c.*3099A>T,XM_011514279.3:c.*3099A>T	.	.	0/1	0/0
chr6	38171889	38171889	T	A	31.93	UTR3	BTBD9	NM_001172418.1:c.*3096A>T,NM_152733.2:c.*3096A>T,NM_052893.2:c.*3096A>T,NM_001099272.2:c.*3096A>T,XM_011514279.3:c.*3096A>T	.	.	0/1	0/0
chr6	42050484	42050484	G	C	49.16	upstream	TAF8	.	.	.	0/1	0/0
chr6	42050489	42050489	G	C	46.16	upstream	TAF8	.	.	.	0/1	0/0
chr6	42050491	42050491	G	C	46.16	upstream	TAF8	.	.	.	0/1	0/0
chr6	42050492	42050492	A	C	46.16	upstream	TAF8	.	.	.	0/1	0/0
chr6	42979882	42979882	C	G	1456.13	UTR5	PEX6	XM_011514661.2:c.-732G>C	.	.	0/1	1/1
chr6	44830880	44830880	G	T	1532.17	ncRNA_exonic	LOC101929770	.	.	.	0/1	1/1
chr6	70271654	70271654	C	A	494.13	splicing	COL9A1	NM_001377289.1:exon8:c.414+1G>T,NM_078485.4:exon8:c.414+1G>T,NM_001377290.1:exon8:c.414+1G>T,XM_017010246.2:exon11:c.594+1G>T,XM_011535429.3:exon14:c.1143+1G>T,NM_001851.6:exon14:c.1143+1G>T	.	.	0/1	0/0
chr6	87513279	87513279	A	G	970.16	downstream	RARS2,SLC35A1	.	.	.	1/1	0/1
chr6	87513281	87513281	A	G	970.16	downstream	RARS2,SLC35A1	.	.	.	1/1	0/1
chr6	87513283	87513283	A	G	964.16	downstream	RARS2,SLC35A1	.	.	.	1/1	0/1
chr6	89676142	89676142	C	T	423.13	exonic	MDN1	.	nonsynonymous_SNV	MDN1:XM_005248700.3:exon38:c.G6590A:p.R2197Q,MDN1:XM_006715405.3:exon41:c.G7049A:p.R2350Q,MDN1:NM_014611.3:exon77:c.G12605A:p.R4202Q,MDN1:XM_011535635.2:exon77:c.G12314A:p.R4105Q,MDN1:XM_011535636.3:exon77:c.G12605A:p.R4202Q,MDN1:XM_024446382.1:exon77:c.G12605A:p.R4202Q	0/1	0/0
chr6	102070152	102070152	C	T	468.13	downstream	GRIK2	.	.	.	0/1	0/0
chr6	138782955	138782955	A	T	1350.59	UTR3	CCDC28A	NM_001379071.1:c.*611A>T	.	.	0/1	1/1
chr6	159586699	159586699	A	G	456.14	downstream	LOC105378085	.	.	.	0/1	0/0
chr6	170553212	170553212	G	C	3402.13	exonic	PSMB1	.	nonsynonymous_SNV	PSMB1:NM_002793.4:exon1:c.C31G:p.P11A	1/1	0/1
chr7	3158628	3158628	A	C	881.14	upstream	LOC100129603	.	.	.	0/1	0/0
chr7	3158652	3158652	T	C	1031.14	upstream	LOC100129603	.	.	.	0/1	0/0

chr7	6955526	6955526	C	T	444.13	UTR3	LOC112267992	XM_024447030.1:c.*43G>A	.	.	0/1	0/0
chr7	22874637	22874637	A	C	271.14	ncRNA_exonic	LOC107986776	.	.	.	0/1	0/0
chr7	56409082	56409082	C	A	1436.16	upstream	LOC101930109	.	.	.	1/1	0/1
chr7	80676955	80676955	G	A	3039.13	UTR3	CD36	NM_001371075.1:c.*572G>A,XM_024447003.1:c.*848G>A,NM_001371077.1:c.*572G>A,NM_001001548.2:c.*572G>A,XM_005250715.5:c.*848G>A	.	.	1/1	0/1
chr7	80677034	80677034	C	G	3195.57	UTR3	CD36	NM_001371075.1:c.*651C>G,XM_024447003.1:c.*927C>G,NM_001371077.1:c.*651C>G,NM_001001548.2:c.*651C>G,XM_005250715.5:c.*927C>G	.	.	1/1	0/1
chr7	80677435	80677435	C	T	2720.27	UTR3	CD36	NM_001371075.1:c.*1052C>T,XM_024447003.1:c.*1328C>T,NM_001371077.1:c.*1052C>T,NM_001001548.2:c.*1052C>T,XM_005250715.5:c.*1328C>T	.	.	1/1	0/1
chr7	81577596	81577596	A	T	441.13	ncRNA_exonic	LOC100128317	.	.	.	0/1	0/0
chr7	93360428	93360428	C	A	116.68	UTR3	VPS50	NM_001257998.1:c.*1992C>A,NM_017667.4:c.*1992C>A	.	.	0/1	0/0
chr7	94355364	94355364	C	A	36.18	ncRNA_exonic	LOC112267858	.	.	.	0/1	0/0
chr7	107742381	107742381	A	T	1675.16	downstream	LOC101927974	.	.	.	0/1	1/1
chr7	116343363	116343363	G	A	513.13	ncRNA_exonic	LOC105375463	.	.	.	0/1	0/0
chr7	149102480	149102480	C	A	1947.16	downstream	ZNF425	.	.	.	0/1	1/1
chr8	23432567	23432567	C	T	2840.42	UTR3	ENTPD4	NM_001128930.2:c.*359G>A,NM_004901.5:c.*359G>A	.	.	1/1	0/1
chr8	23573894	23573894	A	G	3564.15	UTR3	SLC25A37	NM_016612.4:c.*2039A>G,NM_001317812.2:c.*2039A>G,NM_001317813.2:c.*2039A>G,NM_001317814.2:c.*2039A>G	.	.	1/1	0/1
chr8	62981874	62981874	C	T	422.13	UTR3	NKAIN3	NM_001304533.3:c.*16467C>T	.	.	0/1	0/0
chr8	83693174	83693174	C	T	258.13	ncRNA_exonic	LOC105375932	.	.	.	0/1	0/0
chr8	90993299	90993299	C	A	1104.82	downstream	PIP4P2	.	.	.	1/1	0/1
chr8	102811023	102811023	T	G	420.09	ncRNA_exonic	GASAL1	.	.	.	0/1	0/0
chr8	119150994	119150994	C	T	382.13	upstream	LOC105375725	.	.	.	0/1	0/0
chr8	124998526	124998526	C	G	42.13	UTR5	SQLE	NM_003129.4:c.-878C>G	.	.	0/1	0/0
chr9	470259	470259	G	A	852.14	upstream	KANK1,LOC112268042	.	.	.	0/1	0/0
chr9	470270	470270	T	A	820.14	upstream	KANK1,LOC112268042	.	.	.	0/1	0/0
chr9	36678414	36678414	C	T	935.18	downstream	MELK	.	.	.	1/1	0/1
chr9	37512448	37512448	C	T	405.13	UTR3	FBXO10	XM_006716754.4:c.*99G>A,XM_017014619.2:c.*99G>A,NM_012166.3:c.*99G>A	.	.	0/1	0/0
chr9	98012440	98012440	A	G	415.13	exonic	ANP32B	.	nonsynonymous_SNV	ANP32B:NM_006401.3:exon6:c.A656G:p.D219G	0/1	0/0
chr9	107566871	107566871	T	C	356.13	ncRNA_exonic	LOC105376205	.	.	.	0/1	0/0
chr9	107999758	107999758	T	C	1088.77	upstream;downstream	LOC107987112;LOC105376211	.	.	.	0/1	1/1
chr9	112869617	112869617	G	T	1239.06	UTR3	SNX30	NM_001012994.2:c.*774G>T	.	.	1/1	0/1
chr9	113377283	113377283	C	A	721.17	upstream	HDHD3	.	.	.	1/1	0/1
chr9	137038271	137038271	C	A	695.16	downstream	C9orf139	.	.	.	1/1	0/1
chr10	5765645	5765645	A	G	2516.14	UTR3	GDI2,TASOR2	XM_017016071.2:c.*361T>C,NM_001494.4:c.*361T>C,NM_001115156.1:c.*361T>C;XM_011519515.3:c.*2613A>G	.	.	1/1	0/1
chr10	6393574	6393574	T	G	1678.18	UTR3	PRKCQ	XM_005252497.4:c.*809A>C	.	.	1/1	0/1
chr10	13300955	13300955	T	C	41.13	upstream	PHYH	.	.	.	0/1	0/0
chr10	13300961	13300961	G	A	38.13	upstream	PHYH	.	.	.	0/1	0/0
chr10	26741109	26741109	A	G	84.6	UTR3	PDSS1	XM_024447922.1:c.*423A>G	.	.	0/1	0/0
chr10	27111459	27111459	G	A	3851.33	UTR3	YME1L1	NM_139312.3:c.*518C>T,NM_014263.4:c.*518C>T,NM_001253866.1:c.*518C>T,XM_011519300.3:c.*518C>T	.	.	1/1	0/1
chr10	27111460	27111460	C	T	3851.33	UTR3	YME1L1	NM_139312.3:c.*517G>A,NM_014263.4:c.*517G>A,NM_001253866.1:c.*517G>A,XM_011519300.3:c.*517G>A	.	.	1/1	0/1
chr10	72060790	72060790	T	C	3927.52	UTR3	SPOCK2	NM_001244950.2:c.*1970A>G,XM_011540404.3:c.*1970A>G,NM_014767.2:c.*1970A>G	.	.	1/1	0/1
chr10	72060796	72060796	G	T	4025.52	UTR3	SPOCK2	NM_001244950.2:c.*1964C>A,XM_011540404.3:c.*1964C>A,NM_014767.2:c.*1964C>A	.	.	1/1	0/1
chr10	72062503	72062503	C	T	2338.82	UTR3	SPOCK2	NM_001244950.2:c.*257G>A,XM_011540404.3:c.*257G>A,NM_014767.2:c.*257G>A	.	.	1/1	0/1
chr10	116853164	116853164	G	A	281.13	UTR5	HSPA12A	XM_005269673.5:c.-18142C>T	.	.	0/1	0/0
chr10	116883213	116883213	T	C	383.13	UTR3	ENO4,SHTN1	XM_011539797.3:c.*1544T>C;NM_001127211.3:c.*3131A>G,NM_018330.7:c.*3342A>G	.	.	0/1	0/0
chr10	119161436	119161436	A	C	1048.27	UTR5	SFXN4	XM_024447793.1:c.-1697T>G	.	.	0/1	1/1
chr11	9572699	9572699	T	A	99.61	upstream	WEE1	.	.	.	0/1	0/0
chr11	17544445	17544445	G	T	336.13	upstream	USH1C	.	.	.	0/1	0/0
chr11	33073737	33073737	T	C	900.47	UTR3	TCP11L1	XM_017017989.2:c.*1061T>C,XM_011520207.2:c.*1061T>C,XM_011520206.2:c.*1061T>C,XM_011520204.2:c.*1061T>C,XM_017017990.1:c.*1061T>C,XM_011520205.2:c.*1061T>C,XM_017017991.1:c.*1061T>C,XM_017017992.1:c.*1061T>C	.	.	0/1	1/1
chr11	33561712	33561712	C	A	301.13	exonic	KIAA1549L	.	nonsynonymous_SNV	KIAA1549L:XM_005252848.3:exon7:c.C3182A:p.T1061N,KIAA1549L:XM_011519969.2:exon7:c.C3182A:p.T1061N,KIAA1549L:XM_017017484.2:exon7:c.C3182A:p.T1061N,KIAA1549L:NM_012194.3:exon8:c.C4055A:p.T1352N,KIAA1549L:XM_011519970.2:exon8:c.C1865A:p.T622N,KIAA1549L:XM_017017486.1:exon8:c.C1865A:p.T622N,KIAA1549L:XM_005252847.3:exon9:c.C3299A:p.T1100N	0/1	0/0
chr11	47426567	47426567	T	G	39.17	upstream	PSMC3	.	.	.	0/1	0/0
chr11	47622705	47622705	T	C	32.69	exonic	MTCH2	.	nonsynonymous_SNV	MTCH2:NM_001317232.1:exon11:c.A794G:p.K265R,MTCH2:NM_001317233.1:exon11:c.A377G:p.K126R,MTCH2:XM_011519960.3:exon11:c.A794G:p.K265R,MTCH2:NM_014342.4:exon12:c.A821G:p.K274R,MTCH2:XM_011519959.2:exon12:c.A821G:p.K274R	0/1	0/0
chr11	56158656	56158656	T	C	1758.62	downstream	OR8K5	.	.	.	0/1	1/1
chr11	61128578	61128578	C	A	2421.82	downstream	CD5	.	.	.	0/1	1/1
chr11	63973756	63973756	G	A	372.13	upstream	COX8A	.	.	.	0/1	0/0
chr11	76349346	76349346	A	C	30.69	downstream	THAP12	.	.	.	0/1	0/0
chr11	76383779	76383779	A	C	1502.12	UTR3	GVQW3	NM_001305225.3:c.*1318A>C	.	.	1/1	0/1
chr11	95150495	95150495	A	G	2059.16	upstream	LOC101929295	.	.	.	0/1	1/1
chr11	107596552	107596552	G	A	1349.85	UTR3	LOC112267904	XM_024448787.1:c.*3904G>A	.	.	1/1	0/1
chr11	118136041	118136041	G	C	948.16	UTR3	SCN4B	NM_001142349.2:c.*986C>G,NM_174934.4:c.*986C>G,NM_001142348.2:c.*986C>G	.	.	1/1	0/1
chr11	120210691	120210691	G	T	278.14	upstream	OAF	.	.	.	0/1	0/0
chr11	121543553	121543553	G	T	162.13	exonic	SORL1	.	nonsynonymous_SNV	SORL1:NM_003105.6:exon13:c.G1691T:p.S564I	0/1	0/0
chr11	124114499	124114499	G	A	95.93	upstream	VWA5A	.	.	.	0/1	0/0
chr11	124114501	124114501	G	A	96.66	upstream	VWA5A	.	.	.	0/1	0/0
chr11	130890667	130890667	T	C	2341.06	UTR3	SNX19	NM_001347920.2:c.*11109A>G	.	.	0/1	1/1
chr12	5011780	5011780	C	A	1523.27	ncRNA_exonic	LOC100507560	.	.	.	1/1	0/1
chr12	19372401	19372401	G	T	1119.15	UTR3	PLEKHA5	NM_001143821.3:c.*1691G>T	.	.	1/1	0/1
chr12	19521977	19521977	A	G	321.13	UTR3	AEBP2	NM_001363736.2:c.*3257A>G,NM_001114176.2:c.*3257A>G,NM_153207.5:c.*3860A>G,NM_001267043.2:c.*3257A>G	.	.	0/1	0/0
chr12	19651924	19651924	G	C	1205.2	ncRNA_exonic	LOC101928387	.	.	.	0/1	1/1
chr12	52899772	52899772	T	A	56.71	intronic	KRT8	.	.	.	0/1	0/0
chr12	52899776	52899776	T	A	54.14	exonic	KRT8	.	nonsynonymous_SNV	KRT8:NM_002273.4:exon5:c.A980T:p.Q327L,KRT8:NM_001256282.2:exon6:c.A1064T:p.Q355L,KRT8:NM_001256293.2:exon6:c.A980T:p.Q327L	0/1	0/0
chr12	55996001	55996001	A	T	534.14	UTR5;UTR3	SUOX;RAB5B	XM_024449167.1:c.-5937A>T,XM_017019905.2:c.-5937A>T,XM_017019907.2:c.-	.	.	0/1	0/0

								6221A>T;NM_001252036.1:c.*3789A>T,NM_001252037.1:c.*3789A>T,NM_002868.4:c.*3789A>T				
chr12	69354054	69354054	C	T	2864.13	UTR3	LYZ	NM_000239.3:c.*835C>T	.	.	1/1	0/1
chr12	95943293	95943293	A	C	61.69	UTR5	CCDC38	XM_011537883.2:c.-6784T>G	.	.	0/1	0/0
chr12	114030391	114030391	G	A	2051.15	ncRNA_exonic	LOC105369993	.	.	.	0/1	1/1
chr12	132471079	132471079	G	C	598.14	downstream	MUC8	.	.	.	0/1	0/0
chr13	19347248	19347248	A	G	2722.77	downstream	LINC00421	.	.	.	1/1	0/1
chr13	19782801	19782801	T	A	2864.14	UTR5	PSPC1	XM_011535139.2:c.-44A>T,NM_001354908.2:c.-44A>T,NM_001363660.2:c.-44A>T,XM_011535137.2:c.-44A>T,XM_011535138.3:c.-44A>T,NM_001354909.2:c.-44A>T,XM_011535142.3:c.-44A>T,XM_011535141.3:c.-44A>T	.	.	1/1	0/1
chr13	20021326	20021326	C	T	1028.53	UTR5	ZMYM2	XM_011535223.2:c.-3999C>T	.	.	1/1	0/1
chr13	24512713	24512713	G	A	255.13	exonic	PARP4	.	nonsynonymous_SNV	PARP4:XM_011534931.1:exon1:c.C266T:p.S89F	0/1	0/0
chr13	34033596	34033596	T	G	1802.56	upstream	LOC107984589	.	.	.	1/1	0/1
chr13	110457323	110457323	A	C	368.14	exonic	COL4A2-AS2	.	nonsynonymous_SNV	COL4A2-AS2:NM_001267044.1:exon5:c.T402G:p.D134E	0/1	0/0
chr14	20244483	20244483	A	T	212.63	downstream	OR11H4	.	.	.	0/1	0/0
chr14	20633093	20633093	C	A	351.13	downstream	TRP-TGG1-1	.	.	.	0/1	0/0
chr14	22767315	22767315	C	T	2990.2	exonic	OXA1L	.	nonsynonymous_SNV	OXA1L:NM_005015.5:exon2:c.C131T:p.A44V	1/1	0/1
chr14	26393747	26393747	C	T	317.13	ncRNA_exonic	LOC105370418	.	.	.	0/1	0/0
chr14	53511428	53511428	G	T	1362.17	ncRNA_exonic	LOC105370504	.	.	.	0/1	1/1
chr14	54685282	54685282	C	G	1898.35	ncRNA_exonic	LOC729451	.	.	.	0/1	1/1
chr14	95345860	95345860	G	T	1684.15	upstream	LOC107984710	.	.	.	0/1	1/1
chr14	95345862	95345862	C	T	1595.15	upstream	LOC107984710	.	.	.	0/1	1/1
chr14	97803083	97803083	T	C	1294.19	downstream	LOC105370651	.	.	.	0/1	1/1
chr15	24088177	24088177	G	C	253.13	downstream	PWRN4	.	.	.	0/1	0/0
chr15	37998441	37998441	G	A	376.13	downstream	TMCO5A	.	.	.	0/1	0/0
chr15	48242355	48242355	G	A	441.13	ncRNA_exonic	LOC107984758	.	.	.	0/1	0/0
chr15	64737026	64737026	A	C	45.37	ncRNA_exonic	LOC107984737	.	.	.	0/1	0/0
chr15	65049338	65049338	G	A	1593.13	UTR5	SLC51B	XM_005254159.5:c.-667G>A	.	.	1/1	0/1
chr15	70642387	70642387	C	T	1252.22	downstream	LOC107984791	.	.	.	1/1	0/1
chr15	74181424	74181424	G	A	335.13	exonic	STRA6	.	nonsynonymous_SNV	STRA6:NM_001142617.2:exon17:c.C1555T:p.P519S,STRA6:NM_001142618.1:exon17:c.C1555T:p.P519S,STRA6:NM_001142619.2:exon17:c.C1528T:p.P510S,STRA6:NM_001199040.1:exon17:c.C1666T:p.P556S,STRA6:NM_001199041.1:exon17:c.C1600T:p.P534S,STRA6:NM_001199042.2:exon17:c.C1672T:p.P558S,STRA6:NM_022369.4:exon17:c.C1555T:p.P519S,STRA6:XM_011521883.1:exon17:c.C1555T:p.P519S,STRA6:XM_011521884.1:exon17:c.C1366T:p.P456S,STRA6:XM_017022478.1:exon17:c.C1603T:p.P535S,STRA6:XM_017022479.1:exon17:c.C1555T:p.P519S,STRA6:XM_017022480.1:exon17:c.C1366T:p.P456S	0/1	0/0
chr15	85795914	85795914	G	A	461.13	upstream	KLHL25	.	.	.	0/1	0/0
chr15	88546613	88546613	G	C	48.14	UTR5	DET1	NM_001144074.3:c.-14908C>G,NM_017996.4:c.-10279C>G,NM_001321594.1:c.-10279C>G	.	.	0/1	0/0
chr15	92163556	92163556	T	A	2269.29	UTR3	SLCO3A1	NM_013272.4:c.*421T>A,XM_005254889.1:c.*163T>A	.	.	0/1	1/1
chr15	96771615	96771615	G	T	180.3	downstream	SPATA8-AS1	.	.	.	0/1	0/0
chr16	10890591	10890591	A	G	165.13	ncRNA_exonic	LOC112267907	.	.	.	0/1	0/0
chr16	28097421	28097421	C	A	1034.09	downstream	XPO6	.	.	.	0/1	1/1
chr16	75207779	75207779	C	A	126.32	upstream	CTRB2	.	.	.	0/1	0/0
chr16	85555824	85555824	C	A	673.43	UTR5	GSE1	XM_005255865.4:c.-503C>A,XM_005255864.4:c.-503C>A	.	.	0/1	1/1
chr16	88430220	88430220	G	T	399.13	exonic	ZNF469	.	nonsynonymous_SNV	ZNF469:XM_017023784.1:exon2:c.G2750T:p.G917V,ZNF469:NM_001367624.2:exon3:c.G2750T:p.G917V	0/1	0/0
chr16	89645966	89645966	A	C	3984.82	exonic	CHMP1A	.	nonsynonymous_SNV	CHMP1A:NM_001083314.4:exon6:c.T671G:p.L224R	1/1	0/1
chr16	89645972	89645972	G	A	3976.06	exonic	CHMP1A	.	nonsynonymous_SNV	CHMP1A:NM_001083314.4:exon6:c.C665T:p.A222V	1/1	0/1
chr16	89973747	89973747	C	G	1461.58	upstream	CENPBD1	.	.	.	1/1	0/1
chr17	531954	531954	T	G	1585.13	UTR3	VPS53	NM_001366254.2:c.*873A>C,NM_001366253.2:c.*873A>C,NM_018289.4:c.*873A>C	.	.	1/1	0/1
chr17	4539345	4539345	A	T	2092.2	UTR3	MYBBP1A	XM_024450536.1:c.*557T>A,XM_011523616.2:c.*70T>A,NM_014520.4:c.*70T>A	.	.	1/1	0/1
chr17	35006811	35006811	A	C	33.3	UTR3	LIG3,RFFL	NM_013975.4:c.*2305A>C;NM_001017368.2:c.*5157T>G	.	.	0/1	0/0
chr17	48830160	48830160	G	A	45.77	upstream	CALCOCO2	.	.	.	0/1	0/0
chr17	77723632	77723632	G	A	480.13	ncRNA_exonic	LINC01987	.	.	.	0/1	0/0
chr18	673016	673016	C	T	3134.74	UTR3	ENOSF1,TYMS	NM_001354067.2:c.*1289G>A,NM_202758.5:c.*1289G>A,NM_001354068.2:c.*1289G>A,NM_017512.7:c.*1289G>A,NM_001354066.2:c.*1289G>A,NM_001318760.2:c.*1289G>A,NM_001354065.2:c.*1289G>A;NM_001354868.2:c.*19C>T,NM_001354867.2:c.*19C>T,NM_001071.4:c.*19C>T,XM_024451242.1:c.*19C>T	.	.	1/1	0/1
chr18	55776954	55776954	G	A	253.13	ncRNA_exonic	LINC01415	.	.	.	0/1	0/0
chr18	57550464	57550464	G	A	2859.17	UTR3	FECH	NM_001371095.1:c.*248C>T,NM_001012515.4:c.*248C>T,NM_000140.5:c.*248C>T,NM_001374778.1:c.*248C>T,NM_001371094.1:c.*248C>T,XM_011525882.2:c.*248C>T,XM_011525881.1:c.*248C>T	.	.	1/1	0/1
chr18	58629571	58629571	A	T	1675.68	upstream	ALPK2	.	.	.	0/1	1/1
chr18	61813280	61813280	A	T	1144.5	UTR3	RNF152	NM_173557.3:c.*2572T>A,XM_011525879.2:c.*2572T>A,XM_011525878.2:c.*2572T>A,XM_017025613.1:c.*2572T>A,XM_005266650.3:c.*2572T>A,XM_017025612.1:c.*2572T>A,XM_005266652.3:c.*2572T>A	.	.	1/1	0/1
chr18	64424462	64424462	G	T	382.13	downstream	LINC01924	.	.	.	0/1	0/0
chr18	70379692	70379692	T	G	423.18	downstream	LINC01910	.	.	.	1/1	0/0
chr19	972821	972821	A	C	868.16	UTR3	ARID3A	XM_024451407.1:c.*756A>C,XM_017026445.1:c.*756A>C,NM_005224.3:c.*756A>C,XM_005259514.4:c.*756A>C,XM_005259513.5:c.*756A>C,XM_017026446.1:c.*756A>C	.	.	0/1	1/1
chr19	2290035	2290035	T	G	47.13	exonic	LINGO3	.	nonsynonymous_SNV	LINGO3:NM_001101391.2:exon2:c.A1742C:p.Q581P	0/1	0/0
chr19	3804703	3804703	T	C	1098.17	UTR3	ZFR2	NM_015174.2:c.*1246A>G	.	.	1/1	0/1
chr19	6582505	6582505	C	G	910.56	UTR3	CD70	NM_001330332.2:c.*795G>C	.	.	0/1	1/1
chr19	7647830	7647830	C	A	31.14	UTR3	STXBP2	NM_001127396.3:c.*20C>A,NM_006949.4:c.*20C>A,NM_001272034.2:c.*20C>A	.	.	0/1	0/0
chr19	7647834	7647834	T	A	36.93	UTR3	STXBP2	NM_001127396.3:c.*24T>A,NM_006949.4:c.*24T>A,NM_001272034.2:c.*24T>A	.	.	0/1	0/0
chr19	7647838	7647838	T	A	38.93	UTR3	STXBP2	NM_001127396.3:c.*28T>A,NM_006949.4:c.*28T>A,NM_001272034.2:c.*28T>A	.	.	0/1	0/0
chr19	7647841	7647841	C	A	38.69	UTR3	STXBP2	NM_001127396.3:c.*31C>A,NM_006949.4:c.*31C>A,NM_001272034.2:c.*31C>A	.	.	0/1	0/0
chr19	8958975	8958975	G	T	218.13	exonic	MUC16	.	nonsynonymous_SNV	MUC16:NM_024690.2:exon3:c.C17795A:p.P5932H,MUC16:XM_017027489.1:exon3:c.C17795A:p.P5932H,MUC16:XM_017027500.1:exon5:c.C8621A:p.P2874H,MUC16:XM_017027486.1:exon6:c.C17915A:p.P5972H,MUC16:XM_017027487.1:exon6:c.C17915A:p.P5972H,MUC16:XM_017027488.1:exon6:c.C17915A:p.P5972H,MUC16:XM_017027490.1:exon6:c.C17915A:p.P5972H,MUC16:XM_017027491.1:exon6:c.C17915A:p.P5972H,MUC16:XM_017027492.1:exon6:c.C17915A:p.P5972H,MUC16:XM_017027493.1:exon6:c.C17915A:p.P5972H,MUC16:XM_017027494.1:exon6:c.C17915A:p.P5972H,MUC16:XM_017027495.1:exon6:c.C17915A:p.P5972H,MUC16:XM_017027499.1:exon6:c.C8702A:p.P2901H	0/1	0/0
chr19	9093109	9093109	C	T	1569.43	upstream	OR1M1	.	.	.	0/1	1/1

chr19	17343337	17343337	C	T	310.09	downstream	GTPBP3	.	.	.	0/1	0/0
chr19	18575154	18575154	G	T	3988.13	UTR3	UBA52	XM_005260054.2:c.*4G>T,NM_001321017.1:c.*4G>T,XM_005260053.3:c.*4G>T,NM_003333.5:c.*4G>T,NM_001321022.2:c.*4G>T,NM_001033930.3:c.*4G>T,NM_001321019.2:c.*4G>T,NM_001321020.2:c.*4G>T,NM_001321018.2:c.*4G>T,XM_005260052.2:c.*4G>T,XM_006722871.2:c.*4G>T,XM_017027198.1:c.*4G>T,NM_001321021.1:c.*4G>T	.	.	1/1	0/1
chr19	20041710	20041710	G	T	1469.54	downstream	LOC105372309	.	.	.	1/1	0/1
chr19	23559090	23559090	A	G	1896.12	downstream	LOC105372338	.	.	.	1/1	0/1
chr19	37391576	37391576	A	T	613.48	UTR3	ZNF527	XM_005259328.5:c.*1697A>T,XM_017027381.1:c.*1697A>T,NM_032453.2:c.*1697A>T,XM_005259329.4:c.*1697A>T,XM_017027380.1:c.*1697A>T	.	.	0/1	1/1
chr19	37391580	37391580	A	T	640.16	UTR3	ZNF527	XM_005259328.5:c.*1701A>T,XM_017027381.1:c.*1701A>T,NM_032453.2:c.*1701A>T,XM_005259329.4:c.*1701A>T,XM_017027380.1:c.*1701A>T	.	.	0/1	1/1
chr19	37467346	37467346	C	T	454.13	UTR5	ZNF569	XM_017026380.1:c.-53166G>A,XM_017026376.1:c.-22425G>A,XM_006723047.4:c.-12484G>A,XM_011526538.3:c.-12484G>A,XM_006723046.2:c.-12484G>A,XM_017026379.1:c.-53166G>A,NM_001330482.2:c.-53166G>A,NM_152484.3:c.-22425G>A	.	.	0/1	0/0
chr19	37695524	37695524	C	A	103.13	downstream	ZNF607	.	.	.	0/1	0/0
chr19	40666153	40666153	G	T	324.91	UTR3	NUMBL	NM_001289980.2:c.*1315C>A,NM_004756.5:c.*1315C>A,NM_001289979.2:c.*1315C>A	.	.	1/1	0/0
chr19	44612345	44612345	C	T	345.13	upstream	IGSF23	.	.	.	0/1	0/0
chr19	49461720	49461720	C	G	2220.62	exonic	ALDH16A1	.	nonsynonymous_SNV	ALDH16A1:NM_001145396.2:exon6:c.C679G:p.L227V,ALDH16A1:NM_153329.4:exon6:c.C679G:p.L227V,ALDH16A1:XM_011526441.1:exon6:c.C592G:p.L198V,ALDH16A1:XM_011526442.1:exon6:c.C592G:p.L198V	1/1	0/1
chr19	49461874	49461874	G	A	2987.12	intronic	ALDH16A1	.	.	.	1/1	0/1
chr19	49908960	49908960	C	G	3034.52	exonic	NUP62	.	nonsynonymous_SNV	NUP62:NM_001193357.2:exon2:c.G848C:p.S283T,NUP62:NM_012346.5:exon2:c.G848C:p.S283T,NUP62:NM_153718.4:exon2:c.G848C:p.S283T,NUP62:NM_016553.5:exon3:c.G848C:p.S283T,NUP62:NM_153719.4:exon3:c.G848C:p.S283T	1/1	0/1
chr19	51458965	51458965	C	T	307.13	upstream	SIGLEC8	.	.	.	0/1	0/0
chr19	52154051	52154051	T	A	1298.17	UTR3	ZNF836	XM_017026389.2:c.*821A>T,XM_011526558.3:c.*821A>T,XM_011526559.3:c.*821A>T	.	.	1/1	0/1
chr20	23376691	23376691	A	G	3438.06	UTR3	NAPB	NM_001283018.1:c.*685T>C,NM_001283026.1:c.*685T>C,NM_001283020.1:c.*685T>C,XM_017028008.1:c.*685T>C,XM_011529313.1:c.*685T>C,NM_022080.3:c.*685T>C	.	.	0/1	1/1
chr20	25965815	25965815	C	A	495.13	ncRNA_exonic	LINC01733	.	.	.	0/1	0/0
chr20	32857585	32857585	A	T	1273.14	UTR5	EFCAB8	XM_024451885.1:c.-6208A>T,XM_024451882.1:c.-6208A>T,XM_024451883.1:c.-6208A>T	.	.	0/1	1/1
chr20	33704999	33704999	T	G	2075.36	UTR3	PXMP4	NM_007238.5:c.*2707A>C,NM_183397.3:c.*2931A>C	.	.	1/1	0/1
chr20	45704960	45704960	C	A	314.13	exonic	WFDC10B	.	nonsynonymous_SNV	WFDC10B:NM_172131.2:exon1:c.G32T:p.S11I	0/1	0/0
chr21	9995528	9995528	A	C	511.16	downstream	LOC105372733	.	.	.	0/1	0/0
chr21	10524654	10524654	C	A	4442.16	UTR5	TPTE	NM_001290224.2:c.-36506C>A,NM_199260.4:c.-14070C>A,NM_199259.4:c.-14070C>A,NM_199261.4:c.-14070C>A	.	.	1/1	0/1
chr21	23876352	23876352	G	C	251.13	ncRNA_exonic	LOC105372750	.	.	.	0/1	0/0
chr21	29175362	29175362	G	C	2724.42	UTR3	MAP3K7CL	NM_001286634.2:c.*470G>C,NM_001286618.2:c.*470G>C,NM_001286617.2:c.*470G>C,NM_001286622.2:c.*470G>C,NM_001371369.1:c.*470G>C,NM_001371374.1:c.*470G>C,NM_020152.4:c.*470G>C,NM_001286619.1:c.*470G>C,NM_001371376.1:c.*470G>C,NM_001371370.1:c.*470G>C,NM_001371373.1:c.*470G>C,NM_001286620.2:c.*470G>C,NM_001371372.1:c.*470G>C,NM_001371371.1:c.*470G>C,NM_001286624.2:c.*470G>C	.	.	1/1	0/1
chr22	36288308	36288308	T	C	3726.14	exonic	MYH9	.	nonsynonymous_SNV	MYH9:NM_002473.6:exon34:c.A4876G:p.I1626V	1/1	0/1
chr22	38686944	38686944	C	G	2542.13	UTR3	JOSD1	XM_005261878.3:c.*958G>C,NM_001360236.2:c.*958G>C,NM_014876.7:c.*958G>C,NM_001360235.2:c.*958G>C	.	.	1/1	0/1
chr22	39320613	39320613	G	A	291.13	upstream	RPL3	.	.	.	0/1	0/0
chr22	39489072	39489072	T	G	30.69	UTR3	MGAT3	NM_002409.5:c.*123T>G,NM_001098270.1:c.*123T>G	.	.	0/1	0/0
chr22	43835152	43835152	C	G	1305.13	UTR3	SULT4A1	XM_011530120.3:c.*876G>C	.	.	1/1	0/1
chr22	50206552	50206552	C	T	415.13	ncRNA_exonic	LOC105373095	.	.	.	0/1	0/0
chrX	24213698	24213698	T	A	1000.95	UTR3	ZFX	NM_001330327.2:c.*2322T>A,XM_017029799.1:c.*2322T>A,XM_005274592.3:c.*2322T>A,XM_017029792.1:c.*2322T>A,XM_011545581.2:c.*2322T>A,XM_017029791.2:c.*2322T>A,XM_017029790.1:c.*2322T>A,XM_006724513.3:c.*2322T>A,XM_017029800.2:c.*2322T>A,XM_017029798.2:c.*2322T>A,XM_005274591.4:c.*2322T>A,XM_017029797.2:c.*2322T>A,NM_001178086.1:c.*2322T>A,NM_001178084.2:c.*2322T>A,NM_001178095.2:c.*3500T>A,NM_003410.4:c.*2322T>A,NM_001178085.1:c.*2322T>A,XM_017029794.1:c.*2322T>A,XM_017029789.1:c.*2322T>A,XM_011545579.1:c.*2322T>A,XM_017029788.1:c.*2322T>A,XM_024452440.1:c.*2322T>A,XM_017029795.1:c.*2322T>A,XM_017029793.1:c.*2322T>A,XM_017029796.1:c.*2322T>A,XM_011545583.3:c.*2322T>A,XM_005274594.5:c.*2322T>A,XM_017029801.1:c.*2322T>A	.	.	0/1	1/1
chrX	37456502	37456502	G	A	469.16	UTR3	PRRG1	NM_001173490.1:c.*2881G>A,NM_001173489.1:c.*2881G>A,NM_001142395.2:c.*2881G>A,NM_000950.3:c.*2881G>A	.	.	1/1	0/0
chrX	47206074	47206074	C	G	1134.13	exonic	UBA1	.	nonsynonymous_SNV	UBA1:NM_003334.4:exon15:c.C1702G:p.L568V,UBA1:NM_153280.3:exon15:c.C1702G:p.L568V,UBA1:XM_017029777.1:exon15:c.C1855G:p.L619V,UBA1:XM_017029779.2:exon15:c.C1720G:p.L574V,UBA1:XM_017029780.1:exon15:c.C1702G:p.L568V,UBA1:XM_017029781.1:exon15:c.C1702G:p.L568V,UBA1:XM_005272649.1:exon16:c.C1720G:p.L574V,UBA1:XM_011543954.2:exon16:c.C1744G:p.L582V,UBA1:XM_017029778.2:exon16:c.C1786G:p.L596V	1/1	0/1
chrX	73005386	73005386	T	C	1620.13	UTR3	PABPC1L2B	NM_001042506.2:c.*1141T>C	.	.	1/1	0/1
chrX	73005391	73005391	T	G	1765.13	UTR3	PABPC1L2B	NM_001042506.2:c.*1146T>G	.	.	1/1	0/1
chrX	73005397	73005397	C	A	1723.13	UTR3	PABPC1L2B	NM_001042506.2:c.*1152C>A	.	.	1/1	0/1
chrX	78127739	78127739	G	C	1190.55	UTR3	PGK1	NM_000291.4:c.*1909G>C	.	.	1/1	0/1
chrX	78130251	78130251	C	T	1500.14	UTR3	TAF9B	NM_015975.5:c.*1359G>A	.	.	1/1	0/1
chrX	81297730	81297730	C	T	1377.13	UTR3	SH3BGRL	XM_011531014.1:c.*503C>T,NM_003022.3:c.*503C>T,XM_011531013.1:c.*503C>T	.	.	1/1	0/1
chrX	119469881	119469881	T	G	1291.13	exonic	SLC25A5	.	nonsynonymous_SNV	SLC25A5:NM_001152.5:exon2:c.T332G:p.L111R	1/1	0/1

Supplementary Table S3. Teratoma formation assay

Cell Types (cell number)	Animal model (transplantation site, period)	Number	Teratoma forming percentage
hPSC (1×10^5)	SCID Beige mice (cervical subcutaneous or intramuscular, 4-6 weeks)	20	100%
EnSC (1×10^7)	SCID Beige mice (cervical subcutaneous or intramuscular, 6 months)	20	0
E-islet (1000 IEQ)	SCID Beige mice (kidney capsule, 4-6 months)	20	0
	NCG-hIL15 humanized mice (kidney capsule, 4 weeks)	6	0
E-islet (6000 IEQ)	Monkey (intrahepatic, 2 months)	1	0
E-islet (30000 IEQ)	Monkey (intrahepatic, 2 months)	1	0

Supplementary Table S4. Key laboratory values before and after transplantation

Table S4: Key Laboratory Values before and after Transplantation

Parameter	Baseline	2W	4W	8W	12W	16W	24W	52W	81W	116W	Normal Range
White-cell count (10^9/Liter)	5.2	4.1	5.1	5.3	4.4	5.2	5.5	4.6	3.9	5.6	3.5-9.5
Hemoglobin (g/Liter)	143	140	136	144	134	131	131	125	116	130	130-175
Platelet count (10^9/Liter)	177	194	155	171	151	194	243	192	202	143	125-350
Total Bilirubin (umol/Liter)	13.4	8.3	6.6	8.2	14.1	11.1	11.2	15.2	12.2	8.0	3.4-17.1
Directed Bilirubin (umol/Liter)	2.7	0	1.5	1.7	2.1	1.8	0	3.4	2.6	3.5	0-3.4
Albumin (g/Liter)	38	39.1	39.9	40.7	37.8	41.7	45.9	41.8	34.1	40.9	40-55
Aspartate aminotransferase (U/Liter)	15	20	17	17	14	26	27	23	21	17	15-40
Alanine aminotransferase (U/Liter)	10	30	13	11	12	23	21	19	17	23	9-50
Creatinine (umol/Liter)	87	84	90	77	77	72	60	87	82	103	57-111
estimated GFR (CKD-EPI calculation, mL/min per 1·73 m^2)(ml/min)	84	82	80	94	94	97	-	83	89	72	>60
D-dimer (ug/ml)	0.56	-	-	0.64	1.35	0.29	0.5	0.99	-	-	0-1
CD4+ cell count (%)	26	29.7	27.8	28	31.9	35.8	-	21.3	28.7	-	25.8-41.6
CD8+ cell count (%)	41	37.3	38.1	38.3	39.9	39	-	39.6	40.6	-	18.1-29.6
CD4/CD8 ratio	0.63	0.8	0.73	0.73	0.8	0.92	-	0.43	0.71	-	1.57-2.93
Insulin specific antibodies*	Neg	-	-	-	Neg	Neg	-	-	-	-	Neg
FK506 concerntration (ng/ml)	3.6	4.5	3.7	3.9	3	5.9	-	5.9	4.7	5.2	5-10

* Included IA-2A, Zn-8, IAA, GADA, and ICA-40KDA, ICA-64KDA.

Supplementary Table S5. Primary follow-up objectives

Table S5: Primary follow-up objectives

Parameter	Baseline	2W	4W	8W	12W	16W	24W	36W	52W	81W	105W	Normal Range
					Mixed-meal Tolerance Test							
Fasting serum glucose concentration (mmol/L)	3.9	13	6.8	5.4	6.4	6	5.6	4.75	3.5	5.2	5.0	3.9-6.1
15min serum glucose concentration (mmol/L)	4.9	7.2	5.2	5.7	6.6	5.9	6.4	5.21	4.1	4.8	5.4	3.9-6.1
30min serum glucose concentration (mmol/L)	5.9	19.5	6.3	7.7	8.7	5.9	6.8	5.64	4.3	4.8	5.1	＜10.5
60min serum glucose concentration (mmol/L)	14.3	8.3	13.1	17.1	16.7	9.4	9.8	5.96	4.9	9.0	7.3	＜10
120min serum glucose concentration (mmol/L)	21.3	6.7	15.3	21.6	22.2	11.8	5.2	7.51	7.3	6.2	9.1	＜7.8
180min serum glucose concentration (mmol/L)	19	5.3	12	17.8	16.8	9.5	6.6	6.61	8.3	5.4	5.8	＜6.9
240min serum glucose concentration (mmol/L)	-	-	-	12.9	12.5	8.1	5.5	4.75	5.8	3.5	5.1	3.9-6.1
AUC·180min of Glucose	2727	1579	2138	2899	2930	1682	1242	1158	1092	1161	1277	-
Fold change in the AUC from baseline	0	-0.4210	-0.2160	0.0631	0.0744	-0.3832	-0.545	-0.5754	-0.5996	-0.5743	-0.5317	-
Fasting serum C-peptide (nmol/L)	0.23	0.43	0.49	0.47	0.66	0.7	1.09	0.29	0.53	0.68	0.51	0.37-1.47
15min serum C-peptide (nmol/L)	0.33	0.56	0.55	0.41	0.61	0.71	0.96	0.24	0.52	0.59	0.60	0.37-1.47
30min serum C-peptide (nmol/L)	0.35	0.5	0.61	0.54	0.77	0.85	0.97	0.45	0.8	0.62	0.60	-
60min serum C-peptide (nmol/L)	0.74	0.67	1.23	0.8	1.13	1.29	2.26	0.65	0.93	3.3	1.29	-
120min serum C-peptide (nmol/L)	2.15	3.33	1.71	1.91	2.2	2.7	1.56	1.82	2.88	3.56	3.34	-
180min serum C-peptide (nmol/L)	2.33	1.99	2.25	1.9	2.71	2.76	1.73	1.43	5.16	1.96	2.84	-
240min serum C-peptide (nmol/L)	-	-	-	1.8	2.19	2.35	1.07	1.12	3.72	1.24	2.41	0.37-1.47
AUC·180min of C-peptide	246.8	312.5	251.1	229.4	295.6	337.9	291.6	197.3	399.2	449.7	369.3	-
Fold change in the AUC from baseline	0	0.2662	0.0174	-0.0705	0.1977	0.3691	0.1815	-0.2006	0.6175	0.8266	0.4963	-
Fasting serum insulin (pmol/L)	16.84	23.6	16.71	16.64	52.96	48.62	94.33	32.53	41.73	83.79	12.36	18.11-173.43
15min serum insulin (pmol/L)	34.59	58.43	39.95	24.16	40.64	520.3	128.9	-	54.58	61.0	37.56	-
30min serum insulin (pmol/L)	29.74	36.04	46.61	60.86	72.63	38.56	114.4	163.07	138.7	56.32	27.05	-
60min serum insulin (pmol/L)	84.79	62.65	205.7	112	147.3	141.2	573.7	173.45	161.5	1232.0	215.9	-
120min serum insulin (pmol/L)	241.4	663.9	206.1	244.2	296	381	171.4	519.53	721.5	644.3	473.8	-
180min serum insulin (pmol/L)	233.5	138.5	245.8	204.7	406.1	441	298.4	416.71	1383	277.3	212.4	-
240min serum insulin (pmol/L)	-	-	-	132	210	262.8	118	-	469.1	95.1	189.2	18.11-173.43
AUC·180min of Insulin	26619	48673	30770	27690	39212	51481	50267	56858	96300	105363	45512	-
Fold change in the AUC from baseline	0	0.8285	0.1559	0.0402	0.4731	0.9340	0.8884	1.13599	2.6177	2.9836	0.7097	-

Supplementary Table S6. Quality control release criteria of EnSCs and E-islets

Quality control release criteria of EnSCs

Test	Item or method	Release Criteria
Cell morphology	Phase contrast observation	Typical epithelial morphology with clear cell boundary. Dense clones consist of irregular cells with diameter of 3-5 μm.
Cell characterization and purity	Flow cytometry	FOXA1+ ≥ 90%
Genomic stability	Karyotype test	Intact karyotype
	Whole genome sequencing	Devoid of known cancer-related mutations and the lowest overall mutational burden compared to patient PBMC.
Cell viability	Flow cytometry	Live cell proportion >90% before frozen and >60% after thaw.
Pathogen test	Mycoplasma	Undetectable
	Sterility	Undetectable
	Virus	Negative
Safety	Teratoma formation test	No teratoma found.

Quality control release criteria of E-islets

Stage	Test	Method	Release Criteria
PP stage	Cell morphology	Phase contrast observation	Typical epithelial morphology with unclear cell boundary. Cells with diameter of ≤ 3 μm.
	Cell characterization and purity	Flow cytometry	PDX1+ ≥ 60%
	Cell viability	Flow cytometry	Live cell proportion >90%
	Pathogen test	Mycoplasma	Undetectable
		Sterility	Undetectable
		Virus	Negative
		Endotoxin	≤1 EU/mL
EP stage	Cell characterization and purity	Flow cytometry	PDX1+/NKX6-1+ ≥ 60%
	Cell viability	Flow cytometry	Live cell proportion >90%
	Pathogen test	Mycoplasma	Undetectable
		Sterility	Undetectable
		Virus	Negative
		Endotoxin	≤1 EU/mL

E-islet	Morphology	Phase contrast observation	Dense spherical cell mass with unclear cell boundary. Average diameter at 150 μm.
	Cell proportion and purity	Flow cytometry	① Pancreatic lineage: PDX1+ ≥ 85%<br>② Endocrine lineage: CHGA+ ≥ 60%<br>③ Endocrine cell proportion:<br>β cell (C-peptide+) ≥ 40%;<br>α cell (Glucagon+) ≥ 20%;<br>δ cell (Somatostatin+) ≤ 15%<br>④ Untargeted hepatic lineage: AFP+ ≤ 2%
	Cell viability	Flow cytometry	Live cell proportion >90%
	In vitro functionality	Static glucose stimulated insulin (C-peptide) secretion assay	Positive, ≥ 1.5 folds change
	Pathogen test	Mycoplasma	Undetectable
		Sterility	Undetectable
		Virus	Negative
		Endotoxin	≤1 EU/mL

Supplementary Table S7. Primer list

Gene	Sequences (Forward, 5' to 3')	Sequences (Reverse, 5' to 3')
PDX1	GAACGCCACACAGTGCCAAATC	AACGCGCATGGGTCCTTGTAAA
NKX6-1	ACACACTGCTGTGCAACTAAAG	ACTTCTAGTCAGTGTAACTCACGATT
INS	TTTGTGAACCAACACCTGTGCGG	GCGGGTCTTGGGTGTGTAGAAGAA
GCG	TTCCCAGAAGAGGTCGCCATTGTT	CAACCAGTTTATAAAGTCCCTGGCGG
SST	GAGAATGATGCCCTGGAACCTGAAGA	ATTCTTGCAGCCAGCTTTGCGT
TBP	TTGCTGAGAAGAGTGTGCTGGAGATG	CGTAAGGTGGCAGGCTGTTGTT

Supplementary Table S8. Antibody list

Antibodies	Source	Catalog No.
Anti-human Alpha-1-fetoprotein	Agilent	Cat# A0008
Anti-human Albumin	Bethyl	Cat# A80-129
Anti-mouse CD45	Biolegend	Cat# 103106
Anti-human CD45	Biolegend	Cat# 304010
Anti-Chromogranin A	Abcam	Cat# AB68271
Anti-C-peptide	Cell Signaling Technology	Cat# 4593S
Anti-Glucagon	Sigma-Aldrich	Cat# G2654
Anti-FOXA1	Santa Cruz Biotechnology	Cat# sc-101058
Anti-NKX6-1	DSHB	Cat# F55A12
Anti-PDX1	R & D Systems	Cat# AF2419
Anti-Somatostatin	Santa Cruz Biotechnology	Cat# sc-74556
Anti-SOX17	R & D Systems	Cat# BAF1924
Streptavidin APC	BD	Cat# 554067

Alexa Fluor 488 Goat Anti-Mouse IgG2a	Jackson ImmunoResearch Labs	Cat# 115-545-206
Alexa Fluor 647 Donkey Anti-Mouse IgG	Jackson ImmunoResearch Labs	Cat# 715-605-151
Alexa Fluor 488 Donkey Anti-Rabbit IgG	Jackson ImmunoResearch Labs	Cat# 711-545-152
Alexa Fluor 647 Donkey Anti-Goat IgG	Thermo Fisher Scientific	Cat# A-21447
Alexa Fluor 488 Rabbit Anti-Mouse IgG	Jackson ImmunoResearch Labs	Cat# 315-545-003
Normal Goat IgG Biotinylated Control	R & D Systems	Cat# BAF108
Normal Mouse IgG2a	Santa Cruz Biotechnology	Cat# sc-3878
Normal Goat IgG Control	R & D Systems	Cat# AB-108-C
Rabbit IgG Isotype Control	Cell Signaling Technology	Cat# 3900s
Purified Mouse IgG1 Kappa Isotype Control	BD	Cat# 557273
Calcein Blue AM Viability Dye	Thermo Fisher Scientific	Cat# 650855
